# Functional Similarities of Protein-Coding Genes in Topologically Associating Domains and Spatially-Proximate Genomic Regions

**DOI:** 10.3390/genes13030480

**Published:** 2022-03-08

**Authors:** Chenguang Zhao, Tong Liu, Zheng Wang

**Affiliations:** Department of Computer Science, University of Miami, 1365 Memorial Drive, Coral Gables, FL 33124, USA; chenguangzhao@miami.edu (C.Z.); tong.liu@miami.edu (T.L.)

**Keywords:** topologically associating domain, TAD, genome 3D structure, functions of protein-coding genes, graph autoencoder, Hi-C, functional similarity network, gene–gene spatial interaction network

## Abstract

Topologically associating domains (TADs) are the structural and functional units of the genome. However, the functions of protein-coding genes existing in the same or different TADs have not been fully investigated. We compared the functional similarities of protein-coding genes existing in the same TAD and between different TADs, and also in the same gap region (the region between two consecutive TADs) and between different gap regions. We found that the protein-coding genes from the same TAD or gap region are more likely to share similar protein functions, and this trend is more obvious with TADs than the gap regions. We further created two types of gene–gene spatial interaction networks: the first type is based on Hi-C contacts, whereas the second type is based on both Hi-C contacts and the relationship of being in the same TAD. A graph auto-encoder was applied to learn the network topology, reconstruct the two types of networks, and predict the functions of the central genes/nodes based on the functions of the neighboring genes/nodes. It was found that better performance was achieved with the second type of network. Furthermore, we detected long-range spatially-interactive regions based on Hi-C contacts and calculated the functional similarities of the gene pairs from these regions.

## 1. Introduction

Multi-level organizations of the eukaryotic genome are identified to characterize the conformation of chromosomes, such as nucleosomes, topologically associating domains (TADs), chromosomes, and chromosome territories. Chromosomes dynamically interact during transcription, replication, and splicing, which influences the accessibility of genes [1,2]. Approaches like chromosome conformation capture (3C) [3] reveal that insulator proteins like 11-zinc finger protein (CTCF) play a role in connecting chromosome structure with gene expression [4,5,6]. A genome-wide approach named Hi-C [7] can detect genomic loci interactions in the whole genome and probe three-dimensional (3D) architecture, which has been applied to many different cell types [8,9,10,11,12,13,14,15]. TADs, with an average length of ~1 Mbp, are identified as the high-level chromatin structure so that the genomic loci within a TAD frequently interact with each other. In comparison, the gap regions between TADs usually have fewer interactions [7]. TADs are highly conserved across cell types and mammalian genomes, which indicates that the chromatin structures of TADs are stable in evolution [7,16,17]. The insulator binding protein CTCF, housekeeping genes, and transfer RNAs are enriched in TAD boundaries and are considered the key elements to determine the 3D structure of the genomes [5]. Ectopic chromosomal contacts and long-range transcriptional misregulation can occur when TAD boundaries are destroyed, which shows that TADs participate in the coordination of regulating genes [18]. TADs can also coordinate the transcriptions of spatially proximal genes and the zones of enhancer activities via chromatin loops [18,19,20,21,22] and influence some interrelated functions of transcriptional regulation [23].

Based on Hi-C contact maps, it is possible to calculate nuclear distance, capture the conformation of the genome, and find the specific long-range interactions of enhancers, silencers, and insulators [3]. The spatial distance of two pieces of chromosomes can be indicated by the number of Hi-C contacts between them [24]. Cao et al. compared the functional similarities of gene pairs that have spatial interactions and those without an interaction [25]. They found that genes with a large number of interactions are more likely to have similar functions. However, they did not study the functional similarities of gene pairs and Hi-C contacts from the perspective of TADs. Since each TAD is a self-interactive genomic structure, genes in the same TAD may share similar functions.

Furthermore, some long-range chromosomal interactions are retained in different species [26,27], and some long-range interactions affect variegated expression [28]. The question we want to answer is whether functionally similar gene pairs can be found through long-range interactive DNA regions. In this research, the gene functions from long-range and highly interactive regions were analyzed to answer this question.

From the perspective of gene evolution, homologous genes share a common ancestor. Paralogs, also called duplicate genes, are homologous genes separated by a duplication event, and orthologs are homologous genes that descend via a speciation event [29]. Many homologous genes are similar in sequence, and the sequence identity and functional similarity of paralogous genes are positively correlated [30]. Therefore, we removed the duplicated genes when we analyzed the data to find out whether the protein-coding genes in the same TADs or gap regions share similar functions. In this way, if a functional similarity was observed, it would not be caused by duplicated genes but the structural patterns of TADs.

## 2. Results

### 2.1. Functional Similarities of Gene Pairs in the Same TAD and between Different TADs

**Figure 1:**
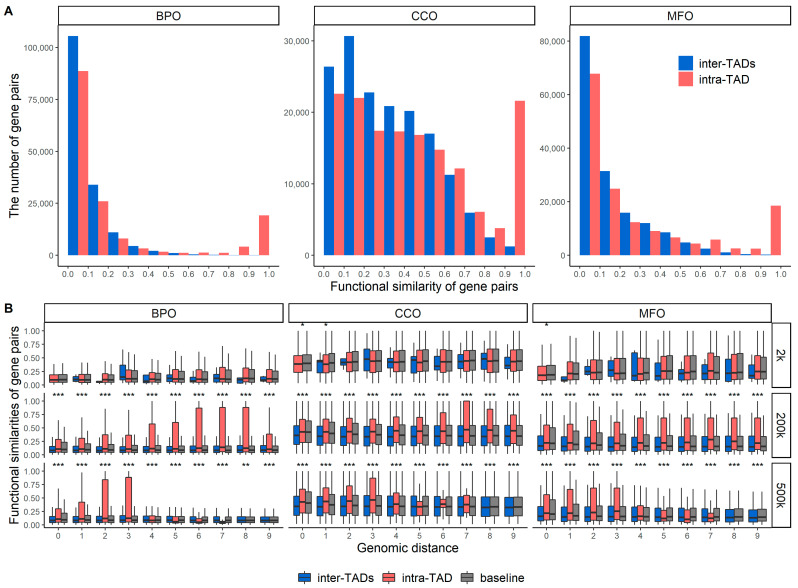
Functional similarities of mouse gene pairs for intra- and inter-TADs. (**A**) depicts the histograms of functional similarities of gene pairs in BPO, CCO, and MFO. The *p*-values of the Wilcoxon rank-sum test are <2.2 × 10^−16^ for all (**A**) plots. (**B**) shows the functional similarities of gene pairs from a range of genomic distances: bin sizes of 2 kbp, 200 kbp, and 500 kbp. The baseline gene pairs were randomly selected regardless of TADs or gaps. *** indicates that the *p*-value of the Wilcoxon test is less than 0.0001, ** indicates that the *p*-value is between 0.0001 and 0.001, and * indicates that the *p*-value is between 0.001 and 0.05.

Figure 1A shows the histograms of functional similarities of mouse gene pairs within the same TADs (intra-TAD) and between different TADs (inter-TADs). We gathered all possible pairwise combinations of intra-TAD gene pairs and then filtered out the gene pairs with protein sequence identities greater than 90% to ensure duplicate genes were removed. We randomly selected the same number of inter-TADs gene pairs and then also removed duplicated genes. After that, more inter-TADs gene pairs were randomly selected and removed so that the number of inter-TADs and intra-TAD gene pairs were the same.

Functional similarities of gene pairs were calculated in terms of three gene ontology (GO) categories: biological process ontology (BPO), cellular component ontology (CCO), and molecular function ontology (MFO) [31]. It can be found that most of the randomly selected gene pairs from intra- and inter-TADs have relatively low similarities. However, many more gene pairs from intra-TADs have very high similarities, which indicates that some genes from the same TADs have very similar or the same functions.

The histograms differ from ontology to ontology. In terms of BPO and MFO, most gene pairs have low similarities, less than 0.4. Functional similarities of some gene pairs are between 0.4 and 0.8, and many intra-TADs gene pairs have high functional similarities that are greater than 0.8. In terms of CCO, functional similarities of many of the gene pairs are less than 0.7, and functional similarities of a significant number of gene pairs are greater than 0.9.

For the histogram of each ontology, we applied the Wilcoxon test and got a *p*-value < 2.2 × 10^−16^, which indicates that the distributions of intra- and inter-TADs are statistically different. These histograms indicate that genes from the same TAD are more likely to have similar or the same functions than those from the different TADs. 

When we randomly selected gene pairs to generate Figure 1A, we selected the gene pairs based on whether they were from the same (intra-) or different (inter-) TADs and whether they were duplicated genes. We did not have any restriction about the genomic distance between the two genes in each gene pair. Therefore, people may argue that the higher functional similarity for intra-TADs may be caused by their shorter genomic distance compared to the gene pairs from different TADs. To address this point, we plotted Figure 1B, in which the genomic distances for gene pairs were unified and grouped into bins. In other words, we compared the functional similarities of intra- or inter-TADs gene pairs with the same genomic distance. 

We tested three bin sizes of genomic distance 2 kbp, 200 kbp, and 500 kbp because these thresholds can make the *X*-axis of Figure 1B cover the majority of the TADs; see Supplementary Figures S1 and S2 for the histograms of the lengths of TADs. For example, when the bin size is 200 kbp, bin 0 on the *X*-axis of Figure 1B indicates that the genomic distances are between 0×200 kbp=0 kbp and 1×200 kbp=200 kbp, and bin 1 indicates that the genomic distances are between 1×200 kbp=200 kbp and 2×200 kbp=400 kbp. The gene pairs in the same TAD or different TADs all have a genomic distance <200 kbp or between 200 kbp and 400 kbp for bins 0 and 1, respectively. 

It is expected to see that when the genomic distance is too big, there are no intra-TAD gene pairs available. It is also expected that when the genomic distance is too small, there are no inter-TAD gene pairs available since the average size of TADs is 1 Mbp. However, our point is to show that, even if we unify the genomic distance, we observe that the protein-coding genes in the same TAD usually have a higher functional similarity than the genes in different TADs. We also observed significant *p*-values for comparing the distributions of intra- and inter-TAD functional similarities in Figure 1B, which are shown in the caption of Figure 1.

Supplementary Figure S3 shows the same type of plots as Figure 1 but without removing duplicate genes. In Supplementary Figure S4, we compared all possible intra-TAD gene pairs and inter-TAD gene pairs from the same chromosome (in Figure 1, the two genes in inter-TAD gene pairs may be from different chromosomes). The findings of these plots are similar to what we have observed in Figure 1, that is, genes in the same TADs usually have higher functional similarities than those from different TADs. 

In Supplementary Figure S5, we applied the same type of analysis as Figure 1 but on a human instead of a mouse. We observed significantly higher functional similarities for intra-TAD genes compared to inter-TADs genes for MFO. For BPO and CCO, higher functional similarities were also observed in intra-TADs genes but not as significant as for MFO. One possible reason for the different functional similarity distributions of mice and humans may be that the gene annotation file that we used for mice contained more unique GO terms than the one that we used for humans (details see the Section 3.3). 

Supplementary Tables S1–S3 show the top 20 enriched gene functions associated with the high functional similarities—that is, >=0.9 similarity scores—from intra-TADs gene pairs in mice (Figure 1A) and humans (Figure S5A). We executed GO enrichment analysis on those intra-TAD gene pairs in BPO, CCO, and MFO, respectively, and observed the following conserved gene functions across mice and humans: ‘detection of chemical stimulus involved in sensory perception of bitter taste’ (GO:0001580), ‘membrane’(GO:0016020), ‘cellular anatomical entity’ (GO:0110165), ‘integral component of membrane’ (GO:0016021), ‘intrinsic component of membrane’ (GO:0031224), ‘chromatin’ (GO:0000785), ‘cell periphery’ (GO:0071944), and ‘bitter taste receptor activity’ (GO:0033038).

### 2.2. Functional Similarities of Gene Pairs in the Same Gap Region and between Different Gap Region

**Figure 2:**
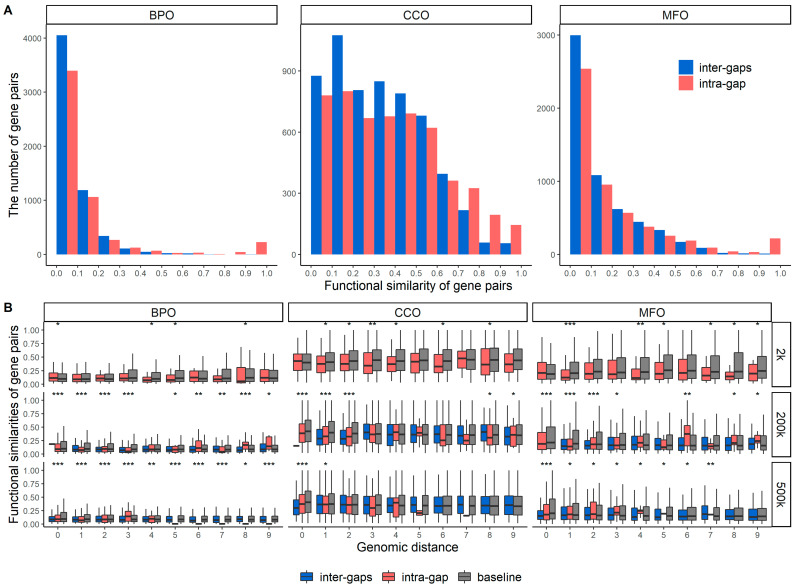
Functional similarities of gene pairs for intra- and inter-gaps. (**A**) depicts the histograms of functional similarities of gene pairs in BPO, CCO, and MFO. The *p*-values of the Wilcoxon rank-sum test for BPO, CCO, and MFO are 1.129 × 10^−15^, 3.083 × 10^−16^, and 2.822 × 10^−6^, respectively. (**B**) shows the functional similarities of gene pairs from a range of genomic distances: bin sizes of 2 kbp, 200 kbp, and 500 kbp. The baseline gene pairs were randomly selected regardless of TADs or gaps. *** indicates that the *p*-value of the Wilcoxon test is less than 0.0001, ** indicates that the *p*-value is between 0.0001 and 0.001, and * indicates that the *p*-value is between 0.001 and 0.05.

A gap region in this research was defined as the intermediate genomic region between two consecutive TADs. We performed the same analysis as in the previous section but for the gene pairs in the same gap region and gene pairs in different gap regions. Figure 2A shows the histograms of functional similarities between the gene pairs of intra- and inter-gap regions. We used all possible combinations of gene pairs for intra-gap analysis and then randomly selected the same number of gene pairs from inter-gaps. The gene pairs that have protein sequence identities greater than 90% were removed. The same numbers of gene pairs from intra- and inter-gaps were used for Figure 2A. 

Although gap regions usually have shorter sequence lengths and contain smaller numbers of genes and fewer internal interactions compared to TADs, these histograms about gap regions also indicate the existence of a group of genes sharing high functional similarities. The *p*-values of the Wilcoxon rank-sum test for Figure 2A BPO, CCO, and MFO subplots are 1.129 × 10^−15^, 3.083 × 10^−16^, 2.822 × 10^−6^, respectively, which indicates that functional similarities of intra- and inter-gaps have different distributions.

TADs are the structural and functional units of the genome. Therefore, it is not surprising to see that the genes in the same TADs share similar functions compared to the genes that are not from the same TADs, as shown in Figure 1. The gap regions are not structural and functional units, but we still observed a similar pattern. The reason for this may be that sequentially nearby genes usually share similar functions, even though they are not from the same TAD. In other words, Figure 2 can also serve as a baseline analysis only showing the effects caused by sequential proximity, whereas Figure 1 shows the effects of both sequential and structural proximities. To quantify that, we applied the Wilcoxon rank-sum test on the distributions shown in Figure 1A and Figure 2A and found that the difference of intra-/inter-TAD distributions is more significant than the difference of intra-/inter-gaps distributions. The *p*-values of the Wilcoxon test for the distributions in Figure 1A and Figure 2A can be found in the captions of the two figures.

Figure 2B shows the functional similarities of gene pairs with three genomic distance units: 2 kbp, 200 kbp, and 500 kbp. For each genomic distance value, we show the distributions of both intra- and inter-gaps and applied the Wilcoxon test on these two distributions. The stars on top of every pair of distributions indicate the level of significance in terms of the *p*-value of the Wilcoxon test. *** indicates a *p*-value of the Wilcoxon test less than 0.0001, ** indicates the *p*-value between 0.0001 and 0.001, and * indicates the *p*-value between 0.001 and 0.05. The same procedures were conducted for the intra- and inter-TAD distributions, as shown in Figure 1B. We can see that the intra-/inter-TAD distributions are much more statistically different than the intra-/inter-gaps distributions. This again demonstrates that the genes located in the same TAD tend to be more functionally similar compared to the genes located in the same gap region. In other words, TAD is a more conservative unit in terms of functions compared to the gap region since the genes located in the same TADs are both sequentially and structurally proximate, whereas the genes in the same gap region are mostly only sequentially proximate.

In Supplementary Figure S6, we show the same analysis as in Figure 2 but without filtering out the gene pairs with >90% protein sequence identity. In Supplementary Figure S7, we used all possible gene pairs for both intra- and inter-gaps on the same chromosome. Similar conclusions can be made on Supplementary Figures S6 and S7 as in Figure 2. 

In Supplementary Figure S8, we applied the same type of analysis of Figure 2 on human genes. More intra-gap genes have high functional similarities (0.9–1.0) compared to inter-gaps genes, particularly for MFO. Moreover, by comparing to Supplementary Figure S5 we can find that the difference (in terms of MFO for similarity 0.9 to 1) between intra- and inter-TADs is bigger than the difference between intra- and inter-gaps, which demonstrates that TADs are more structurally and functionally conserved compared to the gap regions.

### 2.3. Expression Levels of the Gene Pairs in the Same and Different TAD and Gap Region

We defined a score named the ‘gene expression similarity score’ (GESS) to indicate the gene expression similarities between a gene pair (for a definition, see the Section 3.5). A GESS score of 1 indicates that two genes have the same average gene expression level, and 0 indicates that they are largely different.

**Figure 3:**
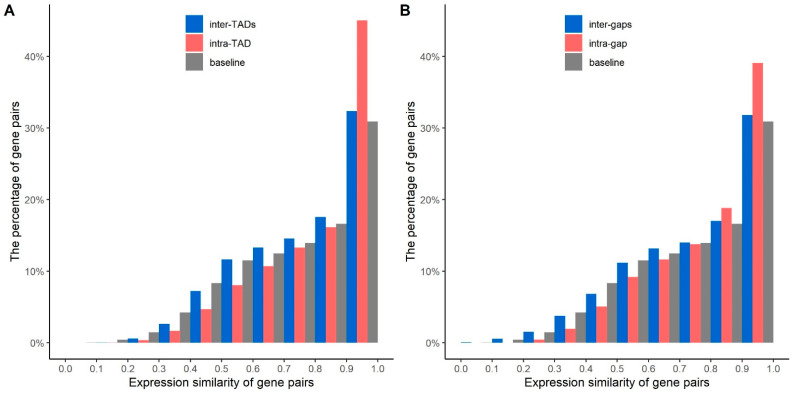
Histograms of expression similarities between gene pairs. (**A**) shows the expression similarities between gene pairs from intra-TAD, inter-TADs, and baseline. (**B**) shows the expression similarities between gene pairs from intra-gap, inter-gaps, and baseline. The baseline indicates that the gene pairs were randomly selected from TADs or gap regions.

Figure 3 shows the histogram of GESS between gene pairs. Figure 3A is for the genes from the same and different TADs, and Figure 3B is for the same or different gap regions. In Figure 3A,B, the baseline gene pairs were randomly picked regardless of whether they were located in the same or different TADs or gaps. The Wilcoxon test *p*-values between any two of the three approaches of selecting gene pairs, inter-TADs, intra-TAD, and baseline, is less than 1.04 × 10^−6^, which indicates that any two distributions are statistically different. 

Notice that when GESS is in the range of approximately 0.9 to 1, intra-TAD gene pairs take a percentage of 45.0% of all gene pairs, which is 1.4 times the percentage value of the inter-TAD gene pairs (32.4%). In comparison, when GESS is in the range of approximately 0.9 to 1, the intra-gap gene pairs take a percentage of 39.1%, which is 1.2 times the percentage value of the inter-gaps gene pairs (31.8%). This shows that the gene expressions of the intra-TAD genes usually are more similar than the gene expressions of the inter-TADs genes, similarly for intra- and inter-gaps, but the difference between intra- and inter-TADs is higher.

We calculated the Pearson’s correlations between GESS (Figure 3) and functional similarities (Supplementary Figures S3 and S6). Table 1 shows the Pearson’s correlations based on three ontologies when the functional similarity threshold is >0.2. The intra-TAD and intra-gap gene pairs achieve the highest correlation values. The correlations of baseline genes are close to those of inter-TADs or inter-gaps gene pairs. 

In summary, we observed high positive correlations between gene expression similarities and gene function similarities for the gene pairs of intra-TAD and intra-gap. In comparison, the correlation values for the inter-TAD and inter-gaps cases are much lower. Moreover, we found that the correlation values for intra-TAD pairs are slightly larger than those of the intra-gap pairs.

Supplementary Table S4 shows the outputs of GO term enrichment analysis for gene pairs that have the mean of normalized expression counts >1000 and expression similarity scores >0.95. With these criteria of the expression counts and the expression similarity score, we found 293, 42, 222, and 12 gene pairs for inter-TADs, inter-gaps, intra-TAD, and intra-gap, respectively. Supplementary Table S5 shows the mutual pathways observed from the same gene pairs listed in Supplementary Table S4. We found four out of 293 inter-TADs gene pairs having mutual pathways (11 mutual pathways) and six out of 222 intra-TAD gene pairs having mutual pathways (34 mutual pathways). The pathway where two genes co-exist was considered a mutual pathway, and one gene pair may have multiple mutual pathways. More gene pairs sharing mutual pathways were found for intra-TAD gene pairs compared to inter-TADs gene pairs.

### 2.4. Functional Similarity Network: The Functional Analysis Based on Network Community 

To further find out how the functionally similar genes co-exist in the same TAD, we constructed a type of biological network of protein-coding genes named functional similarity network (FSN). In an FSN, each node represents a gene, and an edge connecting two nodes indicates that the functional similarity of the two genes is greater than or equal to a similarity threshold, which is set to 0.5.

**Figure 4:**
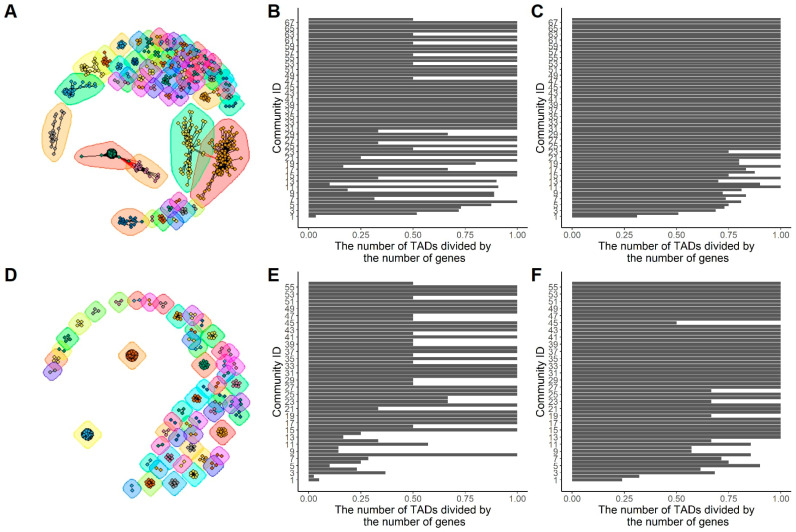
The network communities for BPO FSNs of mouse chromosome 2. (**A**,**D**) show the network communities with functional thresholds 0.7 and 1, respectively. (**B**,**E**) show the distributions of the same-TAD-belonging ratios. (**C**,**F**) show the distributions of the same-TAD-belonging ratios based on random communities.

Figure 4A,D show the network communities detected from the BPO FSNs of the mouse chromosome 2 with functional-similarity-threshold set to 0.7 and 1, respectively. Figure 4B,E list the values of the same-TAD-belonging ratio that is defined as $Num_{same-TAD}/Num_{total}$, where $Num_{same-TAD}$ is the number of TADs that all of the genes in the community belong to, and $Num_{total}$ is the total number of genes in the community. The highest possible value of this ratio is 1, indicating each gene in the community exists in a unique TAD. A smaller ratio indicates more genes exist in the same TAD (s). Figure 4C,F lists the values of the same-TAD-belonging ratio for communities that are randomly selected.

In Figure 4A, 793 genes were clustered into 68 communities. Figure 4B shows the same-TAD-belonging ratios of all of the communities sorted by the number of genes in each community from high to low. For example, community 1 has 286 genes, and community 68 has only two genes. Figure 4C also shows the same-TAD-belonging ratios, but the genes in each community were randomly selected from the FSN. In other words, the same number of genes were randomly selected for each of the communities. The Wilcoxon test *p*-values for the distributions in Figure 4B,C is 0.01, and it is 3.8 × 10^−4^ for the distributions in Figure 4E,F. These *p*-values indicate the statistical significance of the same-TAD-belonging ratios compared to the randomly formed communities.

From these results, we found a trend that large communities are more likely to have lower ratios than small communities. The Pearson’s correlation between the number of genes in the communities and the same-TAD-belonging ratios is −0.39 with *p*-value = 1 × 10^−3^ based on the FSN shown in Figure 4B. From Supplementary Figures S9 and S10, which show the communities detected on CCO and MFO (functional similarity >0.7), we found similar trends, and the correlation values are −0.67 (*p*-value = 2.19 × 10^−48^) for CCO and −0.61 (*p*-value = 1.47 × 10^−0.6^) for MFO, respectively. 

Moreover, as the functional threshold of FSNs was increased from 0.7 to 1, as shown in Figure 4A,D, large communities were gradually disconnected, and we found that more and more genes in the same communities were from the same TAD indicated by a smaller same-TAD-belonging ratio.

We also generated the FSN communities on the mouse X-chromosome, see Supplementary Figures S11–S13, in which similar conclusions can be made as Figure 4.

### 2.5. Gene–Gene Spatial Interaction Network: Graph Reconstruction by a Graph Autoencoder 

We built two types of gene–gene spatial interaction networks to test whether being in the same TAD will improve the ability of graph autoencoders to better learn the topology of the networks and predict edges in the networks. In the first type of gene–gene spatial interaction network, which will be referred to as the HiC-GGSI network, an edge is created between two genes if the number of Hi-C contacts between the two genes is larger than a threshold and the genomic distance between two genes is larger than a certain threshold. For the second type of gene–gene spatial interaction network, which will be referred to as the HiC-TAD-GGSI network, all of the existing edges of HiC-GGSI are kept and if two genes are from the same TAD and the genomic distance between two genes is larger than a certain threshold, a new edge is added. We built both HiC-GGSI and HiC-TAD-GGSI networks for the X-chromosomes and chromosomes 2 (2A for chimpanzee) of the mouse, human, and chimpanzee.

We then applied a state-of-the-art graph autoencoder to the two types of networks. The graph autoencoder contains two components: an encoder and a decoder. The encoder takes a graph (an n×n matrix, with n as the number of nodes) as input and outputs an embedding (an n×p matrix, with p usually smaller than n) in the lower-dimensional space as a representation of the graph. The decoder takes the embedding as input and tries to reconstruct the graph. By comparing the reconstructed graph with the original graph, the algorithm can evaluate and keep improving the abilities of the encoder and decoder. 

For each input graph, we randomly labeled 70% of all edges as training examples, 20% edges as validation examples, and 10% edges as blind test examples. Based on the blind test examples, the area under the curve (AUC) and average precision (AP) were generated to evaluate the accuracy for the graph autoencoder to reconstruct the input graph. 

Table 2 shows the AUC and AP for both HiC-GGSI and HiC-TAD-GGSI networks on the X-chromosomes. Thresholds for Hi-C contacts and genomic distance were applied, which meant that only the gene pairs having ≥threshold Hi-C contacts and ≥threshold genomic distance were used to build the networks. 

In general, the graph autoencoder performed well at reconstructing the input graphs. In particular, the performance with HiC-TAD-GGSI networks is better than the performance with HiC-GGSI networks. These evaluation results indicate the good ability of the encoder to capture the topology of the networks and that including the relationships of being in the same TAD lets the network contain topological patterns that can be better learned by the autoencoder. In other words, if the input is a random graph, the autoencoder usually would have bad performance because the network contains no or few patterns that can be learned.

Supplementary Table S6 shows the AUC and AP of HiC-GGSI and HiC-TAD-GGSI networks on chromosomes 2 of a mouse and a human, and chromosome 2A of a chimpanzee, in which the HiC-TAD-GGSI networks also achieve better performance compared to the HiC-GGSI networks.

To investigate the difference between HiC-GGSI and HiC-TAD-GGSI network topologies, we plotted these two types of networks with Hi-C thresholds 800 and a distance threshold of 2 Mbp on mice. The networks and their topological properties are shown in Supplementary Figures S14 and S15. To compare the topological properties of the original and reconstructed networks, we selected one out of ten runs in Table 2 and plotted the reconstructed networks with their topological properties in Supplementary Figures S16 (input is Supplementary Figure S14) and S17 (input is Supplementary Figure S15). The topological properties include (1) node degree, which is the number of edges that a node has with other nodes over the whole network, (2) the shortest path length, which shows the number of node pairs with various shortest-path values, (3) cluster coefficient, which is a measure of the extent to which a node tends to cluster with other nodes, and (4) closeness centrality, which is a measure of the speed of information spreading from one node to others. 

We observed that the node degree distributions of the original HiC-GGSI, reconstructed HiC-GGSI, and original HiC-TAD-GGSI networks follow a power law, which is the notable characteristic of scale-free networks. In terms of the average shortest path length distribution in Supplementary Figures S14–S17, most nodes are not direct neighbors with others, but they can be reached from most of the other nodes in a few steps, which is a phenomenon of small-world networks.

### 2.6. Gene–Gene Spatial Interaction Network: Functional Inference Based on Reconstructed Networks

The output of the decoder is an n×n matrix, where n is the number of nodes in the input graph. In other words, the decoder assigns a confidence score for every pair of nodes, indicating the probability for each pair of nodes to have an edge. Notice that the decoder assigns these confidence scores based on the embedding or the global topological patterns of the input graph. Therefore, some outlier edges, such as the edges created by mistakes, may have low confidence scores, whereas new edges may be predicted even if the edges do not exist in the input graph. This feature of the graph autoencoder has been used to impute or predict missing edges. Therefore, in our research, we used the reconstructed or imputed graphs to infer the functions of genes based on the topologies of reconstructed networks. 

We used the graph autoencoder to generate reconstructed HiC-GGSI networks and reconstructed HiC-TAD-GGSI networks. For each gene or node in the reconstructed network, we suppose its function is unknown and used the GO terms of its neighboring nodes to infer the function of the central node or gene. Details can be found in the Section 3.9. 

Table 3 shows the performance of functional prediction using the original networks, reconstructed networks, and the combination of original and reconstructed networks on the X-chromosomes of the mouse, human, and chimpanzee. A combination network is the union of the original network and the reconstructed network, that is, it contains all of the edges and nodes existing in the original network and the reconstructed network. 

The highest similarity with the top 1 predicted GO term was calculated between the true GO terms of the target gene and the GO term that was ranked at No.1 in the list of all predicted GO terms. We also reported the highest similarity score when considering the top four GO terms among all predicted GO terms. It can be seen that the accuracies using the HiC-TAD-GGSI network are almost always (with only three exceptions) higher than the accuracies using the HiC-GGSI network, which indicates that including the relationship of being in the same TAD does improve the abilities of the network to incorporate functional similarities. 

We can also observe that using the reconstructed networks almost always (with only two exceptions) results in better performance than using the original networks. The observation indicates that the networks reconstructed by the autoencoder can better infer functions compared to the input networks.

Supplementary Table S7 shows the performance of inferring gene functions on chromosomes 2 of a mouse and a human, and chromosome 2A of a chimpanzee. The performance using HiC-TAD-GGSI networks was also found to be better than the performance using HiC-GGSI networks. Using the reconstructed networks also achieves better performance than using the input networks in most experiment settings. 

### 2.7. Identifying Gene Pairs with Similar Functions from Long-Range Interactive Regions

The long-range (in terms of genomic distance) spatially proximate regions in each mouse chromosome were identified based on the number of intra-chromosomal Hi-C contacts. We retrieved the genes from these interactive regions and then calculated the functional similarities of the gene pairs existing in these regions. 

**Figure 5:**
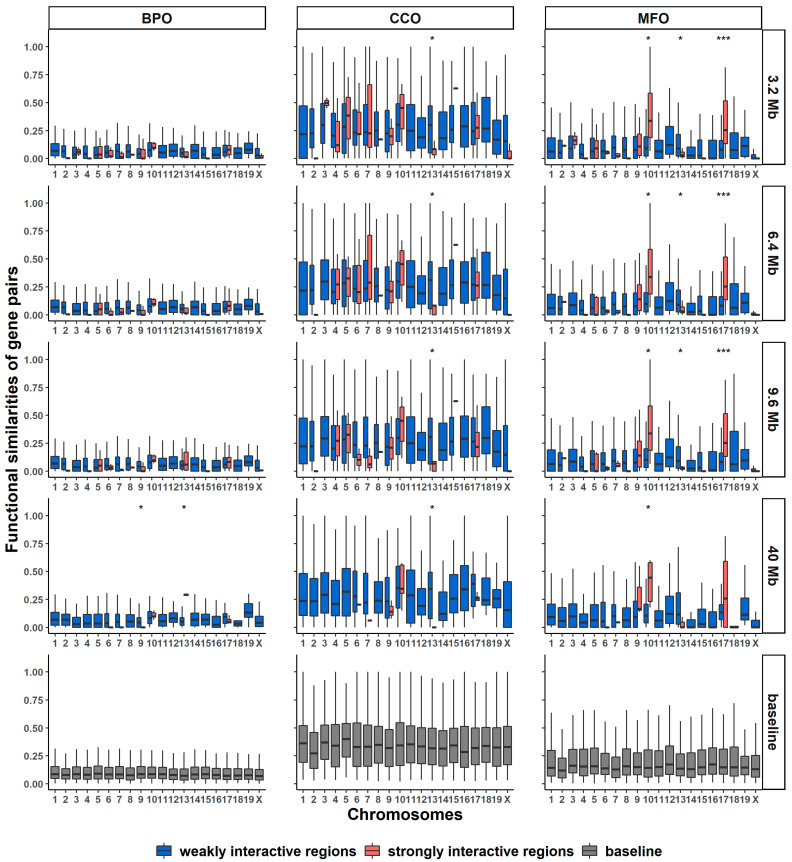
Boxplots of functional similarities of gene pairs from long-range interactive regions in BPO, CCO, and MFO. Different rows represent that the boxplots were generated at different genomic-distance thresholds: 3.2 Mbp, 6.4 Mbp, 9.6 Mbp, 40 Mbp, and baseline. The baseline gene pairs were randomly selected regardless of weakly or strongly interactive regions. *** indicates that the *p*-value of the Wilcoxon test is less than 0.0001, and * indicates that the *p*-value is between 0.001 and 0.05.

Figure 5 shows the functional similarities of the gene pairs identified from the long-range spatially interactive regions. We used two different colors, red and blue, to represent the strongly and weakly interactive regions, respectively, which were defined by two thresholds of Hi-C contacts (details see Section 3.11). To find out how genomic distance affects the distributions of functional similarities, we used four genomic distance thresholds: 3.2 Mbp, 6.4 Mbp, 9.6 Mbp, and 40 Mbp, which means we only used the gene pairs having a genomic distance larger than the thresholds. Since TADs are megabase-sized structures, the threshold values we have tested are all larger than the average size of TADs.

According to the statistics of MFO, when the genomic distance threshold is 3.2 Mb, strongly interactive regions appear in 13 out of 20 chromosomes. We found that the average functional similarities from the strongly interactive regions of chromosomes 10 and 17 are much higher than those of the other chromosomes. As the genomic-distance threshold increases from 3.2 Mbp to 40 Mbp, the number of strongly interactive regions decreases from 13 to 6 out of 20 chromosomes. When the distance threshold is 40 Mbp, the average functional similarities of genes from strongly interactive regions are larger than those from weakly interactive regions in chromosomes 9, 10, and 17. In particular, chromosome 10 has the highest average functional similarities among all chromosomes. Similar patterns can be found for chromosomes 7 and 10 in CCO when genomic distance thresholds are 3.2 Mbp and 9.6 Mbp and chromosome 13 for BPO when genomic distance threshold is 9.6 Mbp. 

For each chromosome, we applied the Wilcoxon test to the functional distributions of both weakly and strongly interactive regions. In Figure 5, *** indicates a *p*-value of the Wilcoxon test less than 0.0001, and * indicates a *p*-value between 0.001 and 0.05. 

**Figure 6:**
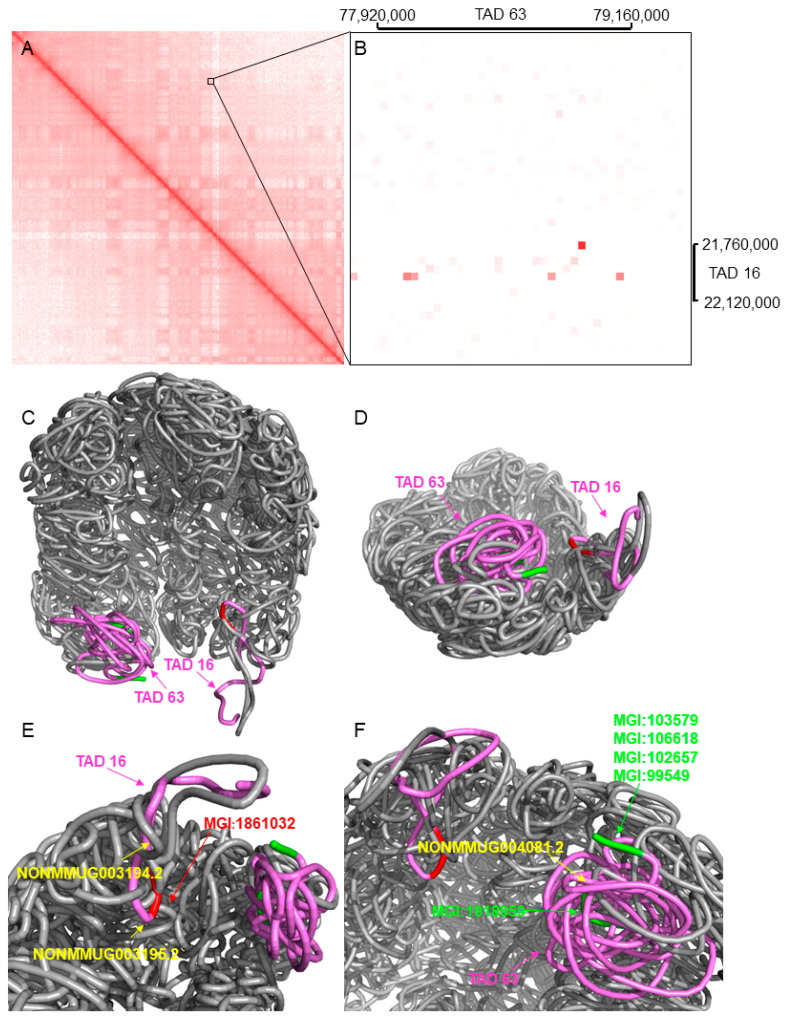
The Hi-C contact map and 3D structure of a long-range spatially interactive region in mouse chromosome 10. (**A**) is the Hi-C contact map of chromosome 10. (**B**) is the zoomed-in Hi-C contact map showing the long-range highly interactive regions containing two TADs. (**C**,**D**) show TADs 16 and 63 from two different perspectives. (**E**,**F**) highlight the gene and lncRNAs in TADs 16 and 63.

To illustrate the details of the genes sharing similar functions in long-range highly-interactive genomic regions, we plotted Figure 6, which is about an example in chromosome 10. Figure 6A illustrates the Hi-C contact map of chromosome 10 at a resolution of 40 kbp. Figure 6B shows long-range highly interactive regions that have functionally similar genes. As shown in a zoomed-in view, we can see that most of the squares with a darker red color are located between two interactive TADs, i.e., TADs 16 and 63. Each square indicates the number of normalized Hi-C contacts between a pair of 40 kbp beads.

To illustrate the scenario with a 3D perspective, we generated the 3D structure of the genomic region starting at TAD 16 and ending at TAD 63 using [24]. Figure 6C–F show the 3D structure of that region from different perspectives. Figure 6C indicates that TADs 16 and 63 are sequentially far away but spatially close to each other. We rotated the 3D model to a different view as in Figure 6D, in which the difference between spatial distance and sequential distance is clearly shown. 

We listed the functionally similar genes and the long non-coding RNAs (lncRNAs) associated with TADs 16 and 63 in Table 4 and highlighted these genes and lncRNAs in Figure 6E,F. Figure 6E focuses on TAD 16, in which the red genomic region indicates gene MGI:1861032, a protein-coding gene, and the yellow arrows point to the locations of lncRNAs NONMMUG003194.2 and NONMMUG003195.2. Figure 6F focuses on TAD 63, in which the green color arrows point to protein-coding genes, and the yellow color arrow points to a lncRNA NONMMUG004081.2. 

The functional similarity between gene MGI:1861032 (retinoic acid early transcript delta) and gene MGI:106618 (tubulin polyglutamylase complex subunit 1) is 0.579, and the functional similarity between gene MGI:1861032 and gene MGI:1918959 (synapse defective 1) is 0.600. All of these genes are annotated with the gene ontology term GO:0005515 protein binding, which indicates that they share similar functions.

In total, we found 39, 33, 25, and 7 gene pairs from long-range highly interactive regions that share similar functions (functional similarity ≥0.5) with genomic distance thresholds 3.2 Mbp, 6.4 Mbp, 9.6 Mbp, and 40 Mbp, respectively. Details of these gene pairs are shown in Supplementary Table S8. 

Supplementary Table S9 shows the results of GO term enrichment analysis for the gene pairs in long-range highly interactive regions. Supplementary Table S10 shows the mutual pathways for some of the gene pairs in long-range highly interactive regions.

We also investigated the functional similarities of the gene pairs that are from different chromosomes. Inter-chromosomal mouse gene pairs were sorted based on the number of raw inter-chromosomal contacts between the genes. In Supplementary Figure S18, we plotted the histograms of functional similarities for the top 100, 1 k, and 50 k gene pairs. We did not find a large number of genes sharing similar functions. Supplementary Table S11 shows the inter-chromosomally interactive gene pairs that exist in the same pathways. From the top 7000 inter-chromosomally interactive gene pairs, we found 14 pairs of genes that have mutual pathways. The total number of mutual pathways for these 14 pairs of genes is 17, as some of the gene pairs have more than one mutual pathway. 

## 3. Materials and Methods

### 3.1. Gene Ontology Definition

The go.obo file defining GO terms and the relationships between GO terms was downloaded from http://geneontology.org/docs/download-ontology/ (accessed on 7 October 2019) [31]. Two versions of go.obo files were used in this work. The first version was released and downloaded on 10 September 2016. It was used for calculating all functional similarities except the evaluation of function prediction based on reconstructed networks (Table 3 and Supplementary Table S7). The second version of the go.obo file was released and downloaded on 7 October 2019. It was only used for calculating GO term similarities in the evaluation of function prediction based on reconstructed networks.

The second version has more GO terms added than the first version. In total, 29,009 biological process terms, 4014 cellular component terms, and 10,297 molecular function terms were defined in the first version go.obo file. The second version has 29,457 biological process terms, 4183 cellular component terms, and 11,093 molecular function terms. 

### 3.2. Calculation of Gene Function Similarity

Each gene is annotated with the GO terms [31] of BPO, CCO, and MFO. We removed the root GO terms for BPO (GO:0008150), CCO (GO:0005575), and MFO (GO:0003674). To precisely calculate the functional similarity of gene pairs, we only considered the GO terms with the following experimental evidence codes: EXP, IDA, IPI, IMP, IGI, and IEP. The calculation of functional similarities between gene pairs was performed with the stand-alone tool GOGO [32]. Functional similarities range from 0 to 1, where 1 indicates that two genes have the same functions, and 0 indicates that two genes have no similar function.

### 3.3. Gene, TAD, and lncRNA Definitions

The definitions of mouse genes were downloaded from the mouse genome informatics (MGI) [33,34,35]. The definitions of TADs on mouse embryonic stem cells were retrieved from [7]. The start and end genomic locations of TADs were defined based on the mouse mm9 reference genome, and the coordinates of genes were defined based on the mouse reference genome mm10. To perform analysis on the same reference genome, we converted the start and end coordinates of TADs from mm9 to mm10 using LiftOver [36]. 

The raw Hi-C contacts data of mice were downloaded from [7] and used to build HiC-GGSI networks.

The TAD and gaps were named sequentially along the DNA sequence of each chromosome. In the TAD definition file, each TAD is defined by the start and end positions in a chromosome. Taking mouse chromosome 5 as an example, the start and end positions of the first two TADs are 3,000,000 to 4,120,000 and 4,200,000 to 49,600,000, respectively. We considered 0 to 2,999,999 as gap 1 and 4,120,000 to 4,200,000 as gap 2. We found that 58.0% and 54.3% of the gaps in humans and mice, respectively, have a genomic length of zero. The gaps that have zero length were not used in our analysis.

The gene body of a gene must entirely reside within a TAD or gap region for us to consider that gene to be existing in that TAD or gap region. The genes that cover more than one TAD or gap or cover a TAD and a gap simultaneously were not considered in this study. In total, we found 17,273 mouse genes existing in 176 TADs and 1649 genes existing in 135 gap regions.

The definitions of human genes were retrieved from the Ensembl database [37]. The definitions of TADs on human embryonic stem cells were retrieved from [7], which were based on the reference genome hg18. We converted human gene definitions from reference genome hg38 to reference genome hg18 using the assembly converter in Ensembl [37]. We found 8757 genes located in 143 TADs and 710 genes located in 143 gap regions.

The raw Hi-C contacts data of humans were downloaded from [7] and used to build HiC-GGSI networks.

The mouse gene annotation file that we downloaded contained 11,349 unique BPO terms, which were 39% of all BPO terms defined in GO. The same file contained 1522 and 3948 unique CCO and MFO terms, which were 38% and 38% of all CCO and MFO terms defined in GO (the first go.obo file), respectively. In comparison, the human gene annotation file that we downloaded contained 6107, 1052, and 2351 unique BPO, CCO, and MFO terms, which were 21%, 26%, and 23% of all the terms defined in GO (the first go.obo file), respectively. The mouse gene annotations that we used covered more GO terms compared to the human gene annotations. 

The definitions and annotations or GO terms of chimpanzee genes were downloaded from the Ensembl database [37]. There are very few experimentally annotated GO terms for chimpanzee genes compared to mice and humans, so we considered all functional annotations (no restriction to the evidence code) of chimpanzee genes. 

The Hi-C reads of chimpanzee lymphocytes were retrieved from [38]. Raw Hi-C reads were processed through the Juicer pipeline [39] and were mapped to human genome assembly hg38 as did in [38]. We then executed LiftOver [36] with default parameters to convert genome coordinates from hg38 to PanTro5. We generated the raw Hi-C contacts using a similar process as in [38]. From the raw Hi-C contacts data, we generated and normalized the 40 kb Hi-C contact matrices with Cooler [40], and then called TADs using hicFindTADs [41].

The definitions of lncRNAs were downloaded from [42].

### 3.4. Removal of Duplicate Mouse and Human Genes

For mouse protein-coding genes, we retrieved protein sequences from the Uniprot database [43]. The mapping relationship between the MGI IDs and SwissProt protein IDs was retrieved from mouse genome informatics [35]. We searched the protein sequences against all the other mouse protein sequences using BLAST [44]. We filtered out the gene pairs with sequence identity scores greater than 90% and an e-value < 0.01. 

For human protein-coding genes, we retrieved DNA sequences from the Ensembl database and then searched the DNA sequences against all the other human DNA sequences using BLAST. We filtered out the gene pairs with sequence identity scores greater than 90% and an e-value < 0.01.

### 3.5. Calculation of the Similarity of Gene Expression Levels

We retrieved the normalized gene expression counts of mouse embryonic stem cells from ESpresso (https://www.ebi.ac.uk/teichmann-srv/espresso/) on 23 March 2017 [45]. For each gene, we calculated the mean of expression counts from cells under different culture conditions. We defined the expression similarity between genes A and B as:

$$\mathrm{GESS}=\frac{\log_{2}\left(\max+1\right)-\log_{2}\left(\min+1\right)-|\log_{2}\left(\mathrm{EC}\left(A\right)+1\right)-\log_{2}(\mathrm{EC}\left(B\right)+1)|}{\log_{2}\left(\max+1\right)-\log_{2}\left(\min+1\right)}$$

where EC(A) and EC(B) are the mean expression counts of genes A and B, respectively, and max and min are the highest and lowest mean expression counts among all genes in the dataset, respectively. We used the log function to convert the original values to smaller values. We added one to each expression count to avoid negative numbers. The expression level similarities range from 0 to 1, with 1 indicating that two genes have the same mean expression level and 0 indicating that they are largely different (the difference is the same as the difference between the maximum and minimum values).

### 3.6. GO Term Enrichment 

We used the web tool PANTHER [46] for GO term enrichment analysis. For each group of genes, we executed BPO, CCO, and MFO enrichment analyses. For each ontology, our result tables show the top three most enriched GO terms. If a GO term and its ancestor GO terms were listed, we only showed the most specific GO term or the GO term that had the largest depth in the GO-directed acyclic graph (DAG). Fisher’s exact test was applied for GO enrichment calculation, and “calculate false discovery rate” was used for result correction.

### 3.7. Mouse Pathway

We downloaded the KEGG pathway [47] for mouse musculus using an R package [48]. We mapped the MGI gene IDs to Ensembl gene IDs and then searched for mutual pathways by checking the mouse musculus pathway map. When two genes co-existed in a pathway, we considered it as a mutual pathway. 

### 3.8. Network Community Detection

Each node in the functional similarity networks indicates a gene, and if the functional similarity between two genes is higher than a threshold, such as 0.7, an edge is created between the two genes. We visualized functional similarity networks using ggnet2 [49] and detected communities in the networks using the cluster_edge_betweenness function [50] in the R package igraph [51]. We chose the default parameters and omitted the weights in the networks. The edges with high edge betweenness centralities are likely to be the boundaries of different communities. We calculated the properties of networks using the plug-in NetworkAnalyzer in Cytoscape [52,53,54]. Random communities were created by randomly selecting the same number of genes as the communities that were detected by igraph. 

### 3.9. Graph Autoencoder

We applied a simple linear graph autoencoder [55] to learn the topologies of HiC-GGSI networks and HiC-TAD-GGSI networks. Compared with the multi-layer graph convolutional network autoencoder, the linear graph autoencoder has fewer parameters to update during training but still achieves competitive performance [55]. We set the learning rate to 0.001, epochs to 200, the number of hidden layers to 2, and the number of hidden units to 128. Since the number of Hi-C reads and the size of the TAD vary across species and chromosomes, we used different Hi-C contact and genomic distance thresholds for different chromosomes. The encoder of the graph autoencoder maps or encodes each node of a graph G to a lower-dimensional space. The decoder reconstructs the graph based on the embedding matrix. 

The encoder is defined as:

$$Z=\tilde{A}W$$

where $\tilde{A}$ is the normalized matrix of the input adjacency matrix *A* with $\tilde{A}=D^{-1/2}AD^{-1/2}$ (*D* is a diagonal matrix with diagonal entries equal to the sum of the corresponding rows of *A*), Z is the low-dimensional space representation, and W is a unique n×d weight matrix to be tuned.

The decoder is defined as:

$$\;\hat{A}=\sigma \left(ZZ^{T}\right)$$

where $\hat{A}$ is the reconstructed matrix, and σ(·) is a logistic sigmoid function. 

An entire graph or network is the only input to the graph autoencoder. The positive or existing edges in the graph were randomly split into three subsets based on the ratio 7:2:1 for training, validation, and blind testing. We also randomly selected two subsets of negative edges (the edges that did not exist in the input graph) for validation and blind testing. The number of negative edges was the same as the number of positive edges. The remaining negative edges are left for training. The output of the autoencoder is a reconstructed n×n adjacency matrix with each entry equaling a value in the range of 0 to 1, indicating the probability that a positive edge exists. 

To evaluate the performance of the graph autoencoder, we used two metrics: average precision (AP) and area under the curve (AUC). The AP and AUC for validation and blind test were calculated between ground truth in *A* and reconstructions in $\hat{A}$. We reported the mean AUC and AP based on 10 repeated experiments as the training, validation, and blind test data were randomly selected for every run. The 95% confidence intervals for the means were generated by the following equation:

$$X\pm t\frac{\sigma }{\sqrt{n}}$$

where X is the mean, t is the degrees of freedom checked from the *t* table, σ is the standard deviation, and *n* is the number of experiments.

### 3.10. Function Inference Based on the Reconstructed Networks

We used a graph autoencoder to generate reconstructed HiC-GGSI networks and reconstructed HiC-TAD-GGSI networks. The graph autoencoder assigned a confidence score to every edge it predicted, and we used a threshold of 0.6 to keep only the edges that had a confidence score higher than that threshold. 

For each gene or node in a reconstructed network, we gathered all of its radius-one neighboring nodes, and for each of the neighboring nodes, we gathered all of its GO terms. Suppose a graph has four genes in total: g1, g2, g3, and g4; gene g1 (the central gene that we do not know the function of) has three radius-one neighboring genes g2, g3, and g4; g2 has three GO terms: GO1, GO2, and GO3; g3 has two GO terms: GO1 and GO4; and g4 has one GO terms: GO1. Additionally, suppose the autoencoder outputs a confidence score of 0.7 for the edge between g1 and g2, a confidence score of 0.6 for the edge between g1 and g3, and a confidence score of 0.8 for the edge between g1 and g4. We used the occurrence of each GO term as the final confidence score for the GO term. For example, the GO term GO1 occurs three times among the neighboring nodes (g2, g3, and g4), so the final score for GO1 is 3. The final score for GO2 would be 1 as it only occurs once (g2). All of the GO terms gathered from the neighboring nodes were ranked based on the final confidence scores. 

When we evaluated the prediction of a gene according to the top 1 predicted GO term, we calculated the semantic similarity between each of its true GO terms and the GO term ranked at No.1 using GOGO [32]. The highest similarity score for each central gene was kept, and the average of the highest similarity scores for all genes (each of these genes was used once as the central gene) was calculated and reported in Table 3. When we evaluated the system according to the top 4 predicted GO terms, each of the top 4 GO terms was compared to each of the true GO terms of the target or central gene, and the highest semantic similarity was kept for the central gene. An average of the highest similarity scores was calculated on all of the genes in the reconstructed network (every node was used as the central gene once). 

### 3.11. Detection of Functionally Similar Gene Pairs from Long-Range Highly Interactive Regions 

We downloaded the normalized Hi-C contact maps of mouse embryonic stem cells from [7], which were based on the mm9 reference genome. We used the Hi-C intra-chromosomal contact maps at a resolution of 40 kbp, which meant each pair of the interactive regions could be considered as a pair of 40 kbp DNA beads. 

We defined thresholds of the number of Hi-C contacts to define strongly and weakly interactive regions. The thresholds needed to allow us to find enough associated genes for comparison. Therefore, strongly interactive regions were defined as a pair of regions having ≥10 normalized Hi-C contacts, and weakly interactive regions were defined as a pair of regions having ≤0.2 but >0 normalized Hi-C contact. 

The genes within a 40 kbp region are defined as all of the genes that have at least one DNA base pair overlapped with the 40 kbp region. For each pair of regions, we gathered all of the genes within each of the two regions and then used the average of the best functional similarity calculated by GOGO [32] to be the functional similarity between the two groups of genes. If the functional similarity between two groups of genes is ≥0.5, we considered them as functionally similar genes and listed the genes in Supplementary Table S8.

## 4. Discussion

By analyzing the gene functions from the perspective of TADs and gap regions, we found that protein-coding genes with similar protein functions tend to cluster in the same TAD or gap region, and they usually share similar expression levels as well. The intra-/inter-TAD distributions are much more statistically different than the intra-/inter-gap distributions, which demonstrates that the genes located in the same TAD tend to be more functionally similar compared to the genes located in the same gap region. In other words, TAD is a more conservative unit in terms of functions compared to the gap region. We constructed functional similarity networks to study the communities of genes and found that many functionally similar genes tend to cluster in the same TAD. 

A graph auto-encoder was applied to learn the topological patterns of the HiC-GGSI and HiC-TAD-GGSI networks and then used to reconstruct the networks. It was found that better performance was achieved with the second type of network, which has the relationship of being integrated in the same TAD. Based on the reconstructed networks, we used the functions of the neighboring nodes to predict the functions of the central nodes and found that the algorithm achieved better performance also with the second type of network. These show that including the relationship of being in the same TAD makes the topological patterns of the gene–gene spatial interaction networks easier to be learned by the graph autoencoder and helps better predict protein functions.

We performed a large-scale analysis for the functions of the genes existing in long-range interactive regions in the same chromosome. We found that the number of strongly interactive regions decreases as the genomic-distance threshold increases. We further observed higher functional similarities between gene pairs in strongly interactive regions than those in weakly interactive regions (chromosomes 9, 10, and 17 in terms of MFO). We further explored an example showing the 3D chromosomal structures of sequentially distant but spatially proximate regions in chromosome 10, which contain genes sharing similar binding functions. The TADs and neighboring lncRNAs were highlighted on the 3D chromosomal structures. 

The definition of TADs can be different based on different TAD-calling methods. Although the method of defining TADs we have applied in this research is one of the first and most widely used methods, in future work we may conduct the same analysis based on different TAD definitions using tools such as Armatus [56], TADtree [57], and HiCExplorer [58].

## 5. Conclusions

The protein-coding genes from the same TAD or gap region are more likely to share similar protein functions, and this trend is more obvious with TADs than the gap regions. Including the relationship of being in the same TAD makes the topological patterns of the gene–gene spatial interaction networks easier to be learned by graph autoencoder and helps better predict protein functions. Gene pairs sharing similar functions do exist in long-range spatially interactive regions. 

## Figures and Tables

**Table 1:** The Pearson’s correlations between functional similarities and expression similarities (functional threshold >0.2). Only two Pearson’s correlations have >0.05 *p*-values (*p*-value listed in parenthesis). All other correlations have <0.05 *p*-values.

	BPO	CCO	MFO
Intra-TAD	0.635	0.423	0.601
Intra-gap	0.586	0.326	0.32
Inter-TADs	0.058	0.052	0.036
Inter-gaps	0.07 (*p*-value: 0.1)	0.099	0.025 (*p*-value: 0.31)
Baseline	0.062	0.029	0.055

**Table 2:** The performance of the graph autoencoder when it was used to reconstruct the HiC-GGSI and HiC-TAD-GGSI networks at different Hi-C contact and genomic distance thresholds on the X-chromosomes of the mouse, human, and chimpanzee. We calculated the 95% confidence interval with ten repeated experiments.

Species	Experimental Settings			Number of Genes in the Network	The Area under the Curve (AUC)	Average Precision (AP)
	Number of Hi-C Contacts between Gene Pairs	Genomic Distance between Gene Pairs	Network Type			
Mouse	≥800	≥1 Mbp	HiC-GGSI	66	0.89 ± 0.069	0.93 ± 0.042
			HiC-TAD-GGSI	230	0.98 ± 0.011	0.99 ± 0.006
		≥2 Mbp	HiC-GGSI	53	0.86 ± 0.096	0.88 ± 0.094
			HiC-TAD-GGSI	71	0.85 ± 0.083	0.9 ± 0.047
	≥1200	≥1 Mbp	HiC-GGSI	58	0.83 ± 0.084	0.84 ± 0.082
			HiC-TAD-GGSI	226	0.98 ± 0.009	0.99 ± 0.01
		≥2 Mbp	HiC-GGSI	48	0.77 ± 0.11	0.84 ± 0.083
			HiC-TAD-GGSI	66	0.86 ± 0.046	0.87 ± 0.059
Human	≥5	≥1 Mbp	HiC-GGSI	197	0.8 ± 0.036	0.86 ± 0.029
			HiC-TAD-GGSI	275	0.94 ± 0.011	0.96 ± 0.006
		≥2 Mbp	HiC-GGSI	167	0.77 ± 0.034	0.81 ± 0.021
			HiC-TAD-GGSI	186	0.83 ± 0.024	0.88 ± 0.021
	≥10	≥1 Mbp	HiC-GGSI	108	0.65 ± 0.077	0.72 ± 0.092
			HiC-TAD-GGSI	203	0.96 ± 0.017	0.97 ± 0.014
		≥2 Mbp	HiC-GGSI	67	0.73 ± 0.076	0.79 ± 0.077
			HiC-TAD-GGSI	86	0.83 ± 0.072	0.86 ± 0.07
Chimpanzee	≥0	≥0.5 Mbp	HiC-GGSI	171	0.8 ± 0.029	0.83 ± 0.016
			HiC-TAD-GGSI	209	0.85 ± 0.021	0.87 ± 0.019
		≥0.7 Mbp	HiC-GGSI	167	0.79 ± 0.026	0.81 ± 0.026
			HiC-TAD-GGSI	177	0.79 ± 0.018	0.82 ± 0.017
	≥5	≥0.5 Mbp	HiC-GGSI	31	0.74 ± 0.16	0.83 ± 0.1
			HiC-TAD-GGSI	88	0.97 ± 0.022	0.97 ± 0.026
		≥0.7 Mbp	HiC-GGSI	25	0.7 ± 0.25	0.81 ± 0.159
			HiC-TAD-GGSI	41	0.82 ± 0.107	0.83 ± 0.096

**Table 3:** Performance of function prediction based on the reconstructed networks on the X-chromosomes of the mouse, human, and chimpanzee. We calculated the 95% confidence interval with ten repeated experiments.

Species	Experimental Settings			GO Terms Considered for Evaluation	Average of the Best Functional Similarity between True GO Terms and the GO Terms Inferred from:		
	Number of Hi-C Contacts between Gene Pairs	Genomic Distance between Gene Pairs	Network Type		Original Network	Reconstructed Network	Union of Original and Reconstructed Networks
Mouse	≥800	≥1 Mbp	HiC-GGSI	Top 1	0.25 ± 0.0	0.27 ± 0.114	0.31 ± 0.078
				Top 4	0.4 ± 0.0	0.48 ± 0.193	0.46 ± 0.087
			HiC-TAD-GGSI	Top 1	0.47 ± 0.0	0.52 ± 0.025	0.51 ± 0.024
				Top 4	0.72 ± 0.0	0.82 ± 0.014	0.8 ± 0.031
		≥2 Mbp	HiC-GGSI	Top 1	0.26 ± 0.0	0.43 ± 0.119	0.29 ± 0.044
				Top 4	0.38 ± 0.0	0.79 ± 0.196	0.41 ± 0.057
			HiC-TAD-GGSI	Top 1	0.45 ± 0.0	0.49 ± 0.155	0.51 ± 0.052
				Top 4	0.68 ± 0.0	0.77 ± 0.216	0.75 ± 0.057
	≥1200	≥1 Mbp	HiC-GGSI	Top 1	0.25 ± 0.0	0.34 ± 0.122	0.27 ± 0.014
				Top 4	0.38 ± 0.0	0.61 ± 0.193	0.42 ± 0.02
			HiC-TAD-GGSI	Top 1	0.48 ± 0.0	0.53 ± 0.014	0.52 ± 0.022
				Top 4	0.73 ± 0.0	0.81 ± 0.013	0.79 ± 0.029
		≥2 Mbp	HiC-GGSI	Top 1	0.27 ± 0.0	0.46 ± 0.08	0.28 ± 0.014
				Top 4	0.39 ± 0.0	0.74 ± 0.098	0.41 ± 0.016
			HiC-TAD-GGSI	Top 1	0.45 ± 0.0	0.45 ± 0.048	0.47 ± 0.031
				Top 4	0.69 ± 0.0	0.8 ± 0.135	0.72 ± 0.045
Human	≥5	≥1 Mbp	HiC-GGSI	Top 1	0.65 ± 0.0	0.79 ± 0.067	0.81 ± 0.053
				Top 4	0.79 ± 0.0	0.89 ± 0.048	0.9 ± 0.04
			HiC-TAD-GGSI	Top 1	0.72 ± 0.0	0.86 ± 0.0	0.86 ± 0.0
				Top 4	0.86 ± 0.0	0.94 ± 0.0	0.94 ± 0.0
		≥2 Mbp	HiC-GGSI	Top 1	0.63 ± 0.0	0.71 ± 0.059	0.84 ± 0.049
				Top 4	0.78 ± 0.0	0.83 ± 0.044	0.9 ± 0.033
			HiC-TAD-GGSI	Top 1	0.68 ± 0.0	0.83 ± 0.0	0.83 ± 0.0
				Top 4	0.82 ± 0.0	0.93 ± 0.001	0.93 ± 0.001
	≥10	≥1 Mbp	HiC-GGSI	Top 1	0.49 ± 0.0	0.63 ± 0.106	0.82 ± 0.08
				Top 4	0.71 ± 0.0	0.79 ± 0.064	0.9 ± 0.048
			HiC-TAD-GGSI	Top 1	0.66 ± 0.0	0.85 ± 0.0	0.85 ± 0.0
				Top 4	0.85 ± 0.0	0.93 ± 0.001	0.93 ± 0.001
		≥2 Mbp	HiC-GGSI	Top 1	0.54 ± 0.0	0.65 ± 0.076	0.72 ± 0.053
				Top 4	0.69 ± 0.0	0.79 ± 0.064	0.85 ± 0.047
			HiC-TAD-GGSI	Top 1	0.58 ± 0.0	0.83 ± 0.0	0.83 ± 0.0
				Top 4	0.77 ± 0.0	0.91 ± 0.003	0.91 ± 0.003
Chimpanzee	≥0	≥2 Mbp	HiC-GGSI	Top 1	0.42 ± 0.0	0.45 ± 0.018	0.49 ± 0.026
				Top 4	0.59 ± 0.0	0.64 ± 0.036	0.68 ± 0.04
			HiC-TAD-GGSI	Top 1	0.44 ± 0.0	0.5 ± 0.027	0.53 ± 0.018
				Top 4	0.62 ± 0.0	0.7 ± 0.028	0.72 ± 0.007
		≥3 Mbp	HiC-GGSI	Top 1	0.41 ± 0.0	0.44 ± 0.024	0.46 ± 0.031
				Top 4	0.58 ± 0.0	0.64 ± 0.036	0.65 ± 0.048
			HiC-TAD-GGSI	Top 1	0.43 ± 0.0	0.51 ± 0.026	0.52 ± 0.019
				Top 4	0.62 ± 0.0	0.71 ± 0.024	0.72 ± 0.005
	≥5	≥2 Mbp	HiC-GGSI	Top 1	0.37 ± 0.0	0.38 ± 0.011	0.18 ± 0.062
				Top 4	0.53 ± 0.0	0.54 ± 0.01	0.36 ± 0.107
			HiC-TAD-GGSI	Top 1	0.4 ± 0.0	0.44 ± 0.036	0.45 ± 0.169
				Top 4	0.58 ± 0.0	0.61 ± 0.024	0.64 ± 0.158
		≥3 Mbp	HiC-GGSI	Top 1	0.42 ± 0.0	0.43 ± 0.004	0.21 ± 0.076
				Top 4	0.61 ± 0.0	0.61 ± 0.005	0.37 ± 0.1
			HiC-TAD-GGSI	Top 1	0.41 ± 0.0	0.44 ± 0.04	0.72 ± 0.201
				Top 4	0.61 ± 0.0	0.62 ± 0.022	0.8 ± 0.148

**Table 4:** The genes and lncRNAs existing in a pair of long-range highly interactive regions. The protein-coding genes in this region share similar functions. In terms of MFO, the functional similarity between MGI:1861032 (retinoic acid early transcript delta) and MGI:106618 (tubulin polyglutamylase complex subunit 1) is 0.579, and the functional similarity between MGI:1861032 and MGI:1918959 (synapse defective 1) is 0.600.

	Genes and lncRNAs
TAD 16	Gene MGI:1861032: retinoic acid early transcript delta
	LncRNA NONMMUG003194.2
	LncRNA NONMMUG003195.2
TAD 63	Gene MGI:1918959: synapse defective 1
	LncRNA NONMMUG004081.2
	Gene MGI:106618: tubulin polyglutamylase complex subunit 1
	Gene MGI:103579: mucosal vascular addressin cell adhesion molecule 1
	Gene MGI:99549: granzyme M
	Gene MGI:102657: cell division cycle 34

## Data Availability

The author confirms that the data supporting the findings of this study are available within the article and Supplementary File.

## Supplementary Materials

The following supporting information can be downloaded at: https://www.mdpi.com/article/10.3390/genes13030480/s1, Figure S1: The histogram of the lengths of TADs of mice; Figure S2: The histogram of the lengths of TADs of humans; Figure S3: Functional similarities of mouse gene pairs for intra- and inter-TADs without removing possible duplicate genes; Figure S4: Functional similarities of all possible mouse gene pairs for intra- and inter-TADs on the same chromosome; Figure S5: Functional similarities of human gene pairs for intra- and inter-TADs removing possible duplicate genes; Figure S6: Functional similarities of mouse gene pairs for intra- and inter-gaps without removing possible duplicate genes; Figure S7: The histograms of percentages of all possible mouse gene pairs for intra- and inter-gaps on the same chromosome; Figure S8: Functional similarities of human gene pairs for intra- and inter-gaps removing possible duplicate genes; Figure S9: The network communities for CCO FSNs of mouse chromosome 2; Figure S10: The network communities for MFO FSNs of mouse chromosome 2; Figure S11: The network communities for BPO FSNs of the mouse X-chromosome; Figure S12: The network communities for CCO FSNs of the mouse X-chromosome; Figure S13: The network communities for MFO FSNs of the mouse X-chromosome; Figure S14: The HiC-GGSI network and topological properties of the mouse X-chromosome with the Hi-C threshold: 800 and the distance threshold: 2 Mbp; Figure S15: The HiC-TAD-GGSI network and topological properties of the mouse X-chromosome with the Hi-C threshold: 800 and the distance threshold: 2 Mbp; Figure S16: The reconstructed HiC-GGSI network and topological properties of the mouse X-chromosome with the Hi-C threshold: 800, the distance threshold: 2 Mbp, and the autoencoder confidence score threshold: 0.6; Figure S17: The reconstructed HiC-TAD-GGSI network and topological properties of the mouse X-chromosome with the Hi-C threshold: 800, the distance threshold: 2 Mbp, and the autoencoder confidence score threshold: 0.6; Figure S18: Functional similarities of highly interactive mouse gene pairs from different chromosomes; Table S1: The top 20 enriched gene functions leading to high functional similarities (≥0.9) of intra-TADs gene pairs of humans and mice in BPO; Table S2: The top 20 enriched gene functions leading to high functional similarities (≥0.9) of intra-TADs gene pairs of humans and mice in CCO; Table S3: The top 20 enriched gene functions leading to high functional similarities (≥0.9) of intra-TADs gene pairs of humans and mice in MFO; Table S4: GO term enrichment analysis for gene pairs with high expression similarities; Table S5: Mutual pathways for gene pairs with high expression similarities; Table S6: The performance of the graph autoencoder when it was used to reconstruct the HiC-GGSI and HiC-TAD-GGSI networks at different Hi-C contact and genomic distance thresholds on the chromosomes 2 of mice and humans and the chromosome 2A of chimpanzees; Table S7: Performance of function prediction based on reconstructed networks on the chromosomes 2 of mice and humans and the chromosome 2A of chimpanzees; Table S8: Functionally similar gene pairs from mouse long-range highly interactive regions with functional similarities >=0.5; Table S9: GO terms enrichment analysis of genes from long-range highly interactive regions; Table S10: Mutual pathways of gene pairs from long-range highly interactive regions; Table S11: The mutual pathways of mouse inter-chromosome interactive gene pairs.
