# Supplementary material for: Functional Similarities of Protein-Coding Genes in Topologically Associating Domains and Spatially-Proximate Genomic Regions
Source: Genes (Basel). 2022 Mar 8;13(3):480. doi: 10.3390/genes13030480 (PMC8951421; doi:10.3390/genes13030480)
Supplement: Supplementary file 1 [file genes-13-00480-s001.zip › genes-1561827-supplementary.pdf]

Supplementary Material of

Functional similarities of protein-coding genes in topologically associating domains and spatially-proximate genomic regions

1 Supplementary Figures

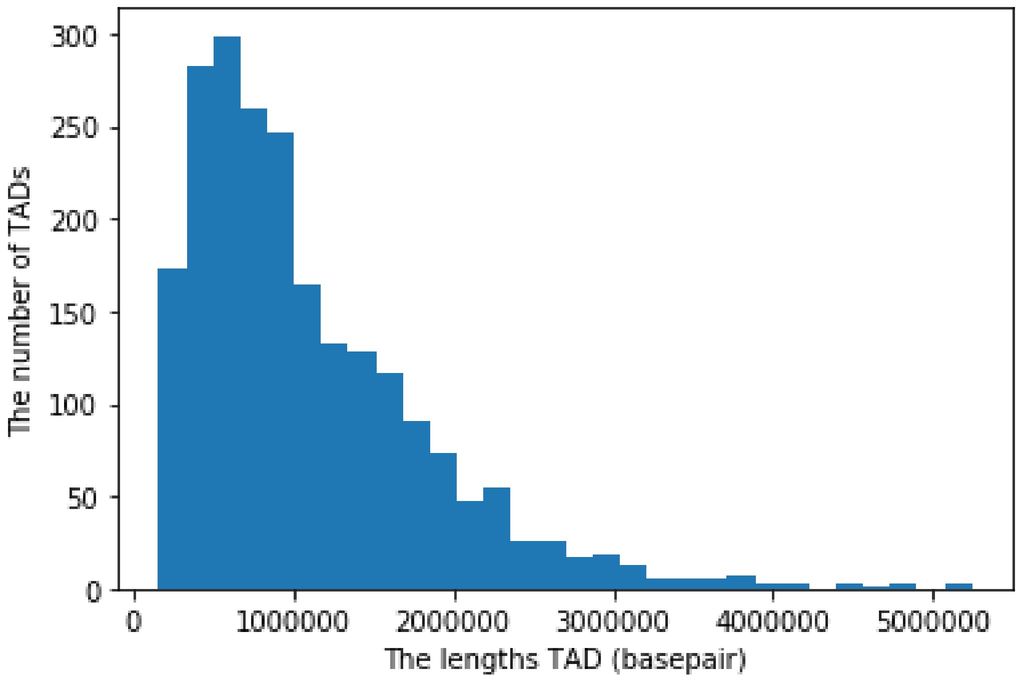

Supplementary Figure S1 The histogram of the lengths of TADs of mice.

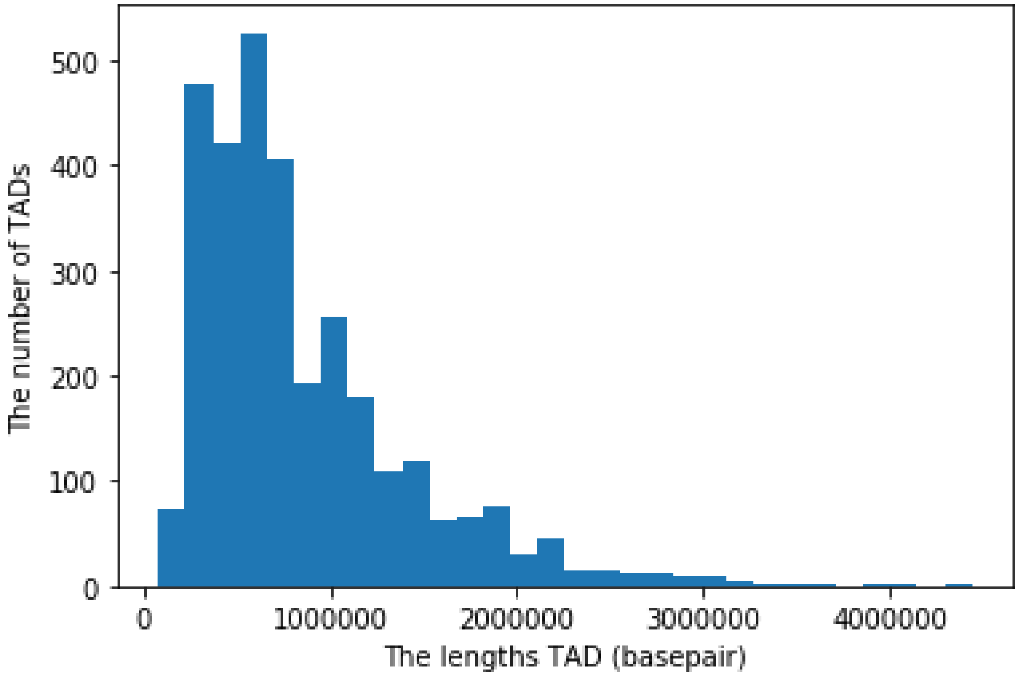

Supplementary Figure S2 The histogram of the lengths of TADs of humans.

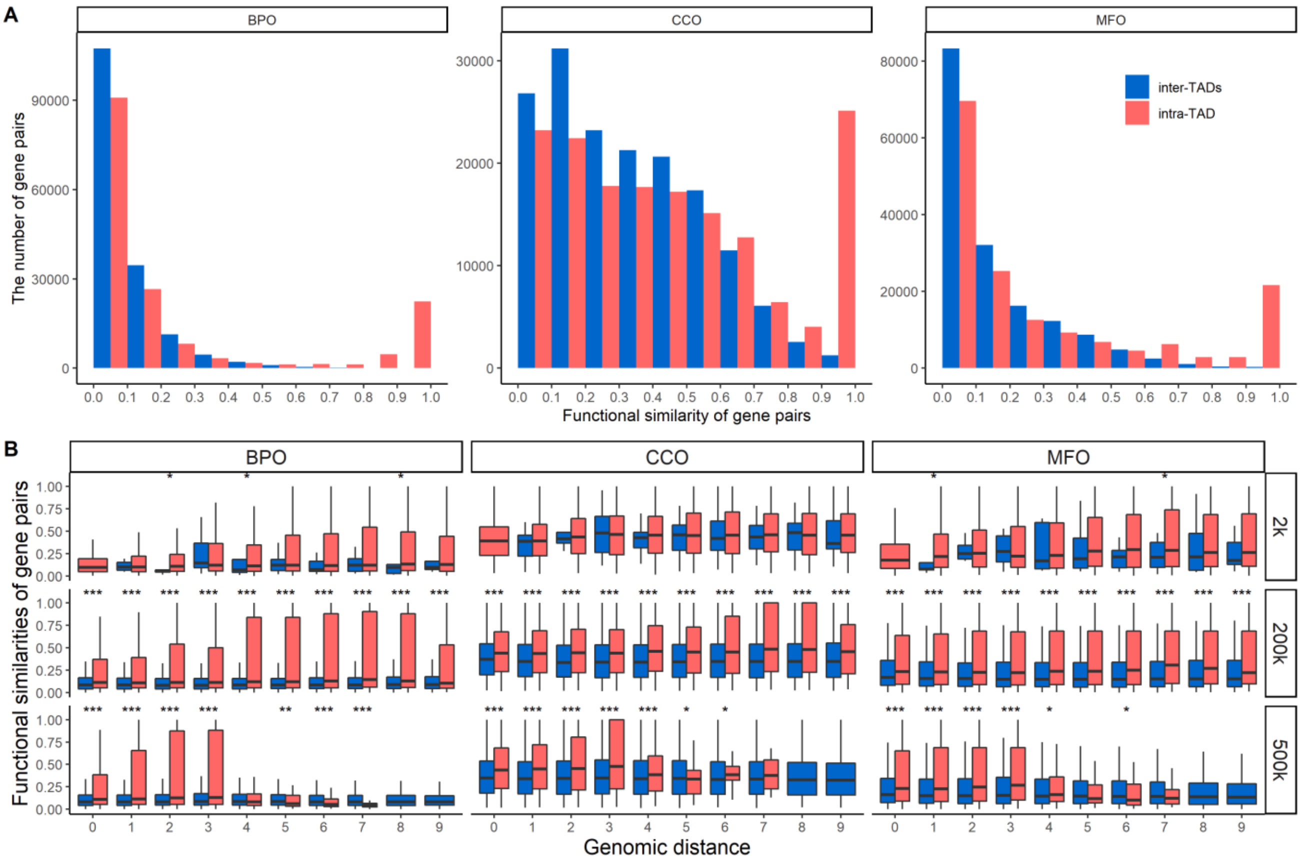

Supplementary Figure S3 Functional similarities of mouse gene pairs for intra- and inter-TADs without removing possible duplicate genes. Supplementary Figure S3A depicts the histograms of functional similarities of gene pairs in BPO, CCO, and MFO. Supplementary Figure S3B shows the functional similarities of gene pairs from a range of genomic distances: bin sizes of 2 kbp, 200 kbp, and 500 kbp. For example, when bin size equals 2 kbp, bin 0 indicates that the genomic distance of the gene pairs is less than 2 kbp. *** indicates that the p-value of the Wilcoxon test is less than 0.0001, ** indicates that the p-value is between 0.0001 and 0.001, and * indicates that the p-value is between 0.001 and 0.05.

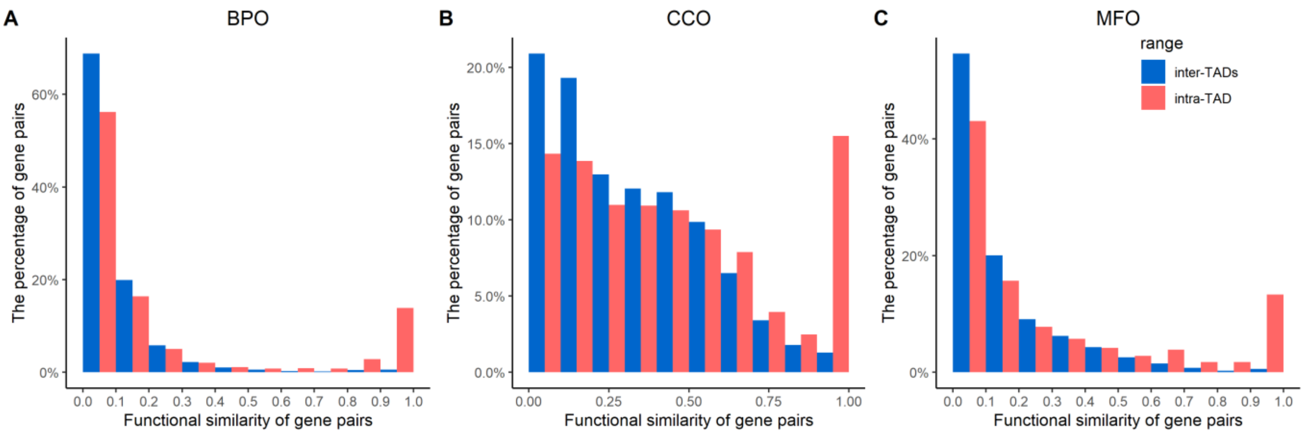

Supplementary Figure S4 Functional similarities of all possible mouse gene pairs for intra- and inter-TADs on the same chromosome. The histograms of functional similarities of gene pairs were calculated in BPO, CCO, and MFO. The Y-axis uses a percentage scale.

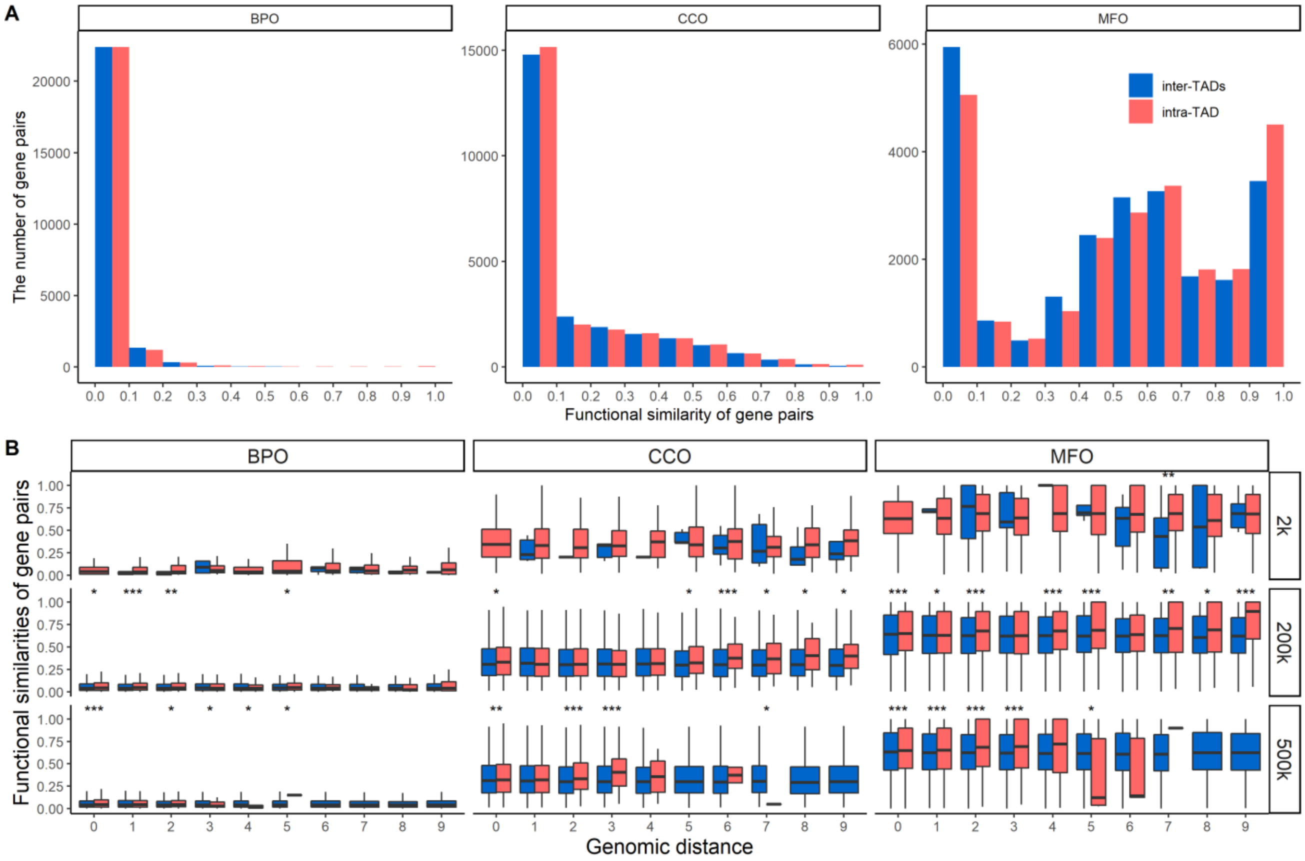

Supplementary Figure S5 Functional similarities of human gene pairs for intra- and inter-TADs removing possible duplicate genes. Supplementary Figure S5A depicts the histograms of functional similarities of gene pairs in BPO, CCO, and MFO. Supplementary Figure S5B shows the functional similarities of gene pairs from a range of genomic distances: bin sizes of 2 kbp, 200 kbp, and 500 kbp. For example, when bin size equals 2 kbp, bin 0 indicates that the genomic distance of the gene pairs is less than 2 kbp. *** indicates that the p-value of the Wilcoxon test is less than 0.0001, ** indicates that the p-value is between 0.0001 and 0.001, and * indicates that the p-value is between 0.001 and 0.05.

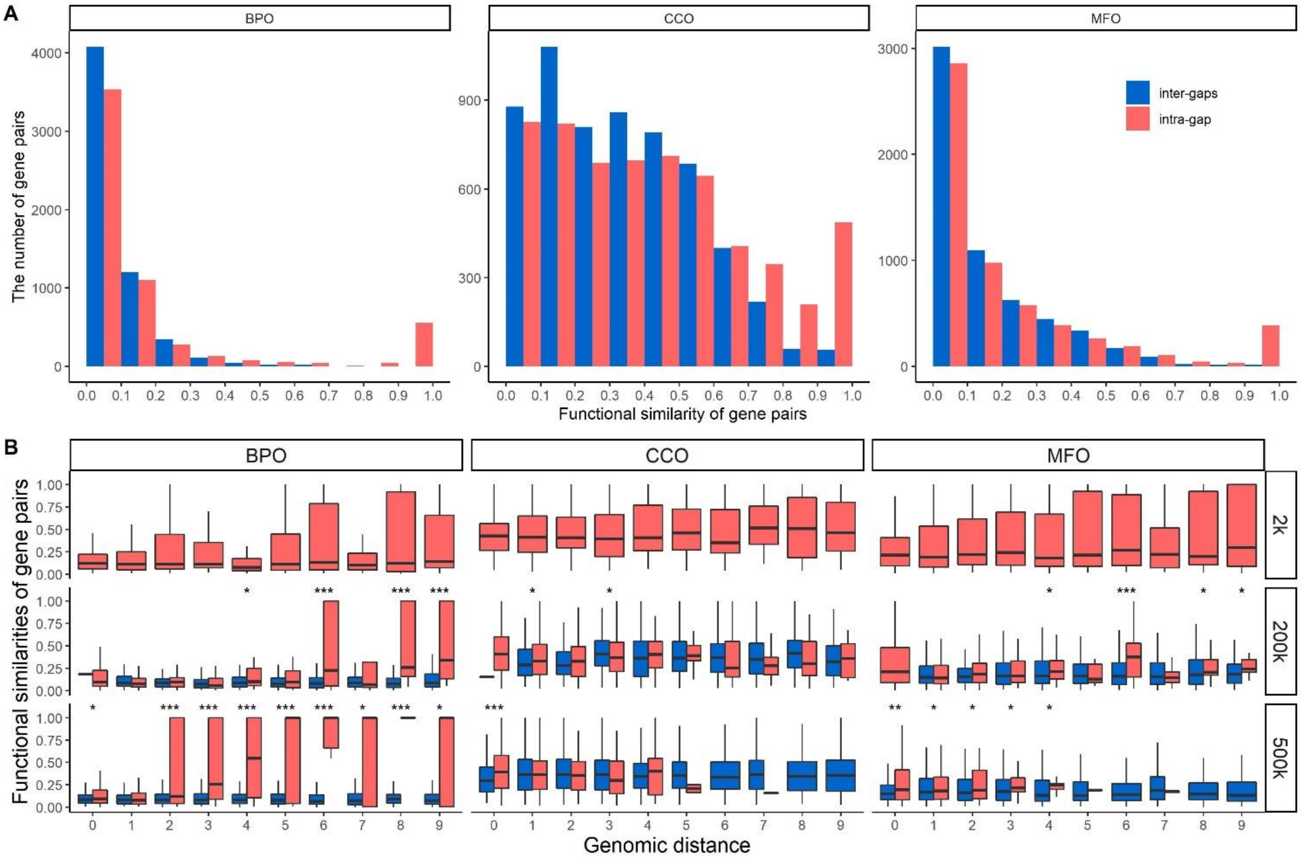

Supplementary Figure S6 Functional similarities of mouse gene pairs for intra- and inter-gaps without removing possible duplicate genes. Supplementary Figure S6A depicts the histograms of functional similarities of gene pairs in BPO, CCO, and MFO. Supplementary Figure S6B shows the functional similarities of gene pairs from a range of genomic distances: bin sizes of 2 kbp, 200 kbp, and 500 kbp. For example, when bin size equals 2 kbp, bin 0 indicates that the genomic distance of the gene pairs is less than 2 kbp. *** indicates that the p-value of the Wilcoxon test is less than 0.0001, ** indicates that the p-value is between 0.0001 and 0.001, and * indicates that the p-value is between 0.001 and 0.05.

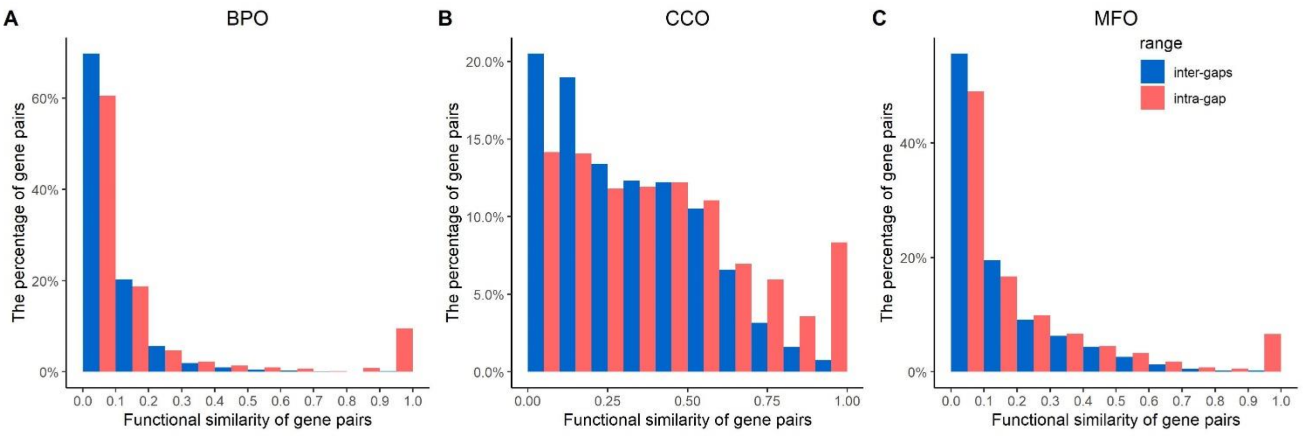

Supplementary Figure S7 The histograms of percentages of all possible mouse gene pairs for intra- and inter-gaps on the same chromosome. The histograms of functional similarities of gene pairs were calculated in BPO, CCO, and MFO. The Y-axis uses a percentage scale.

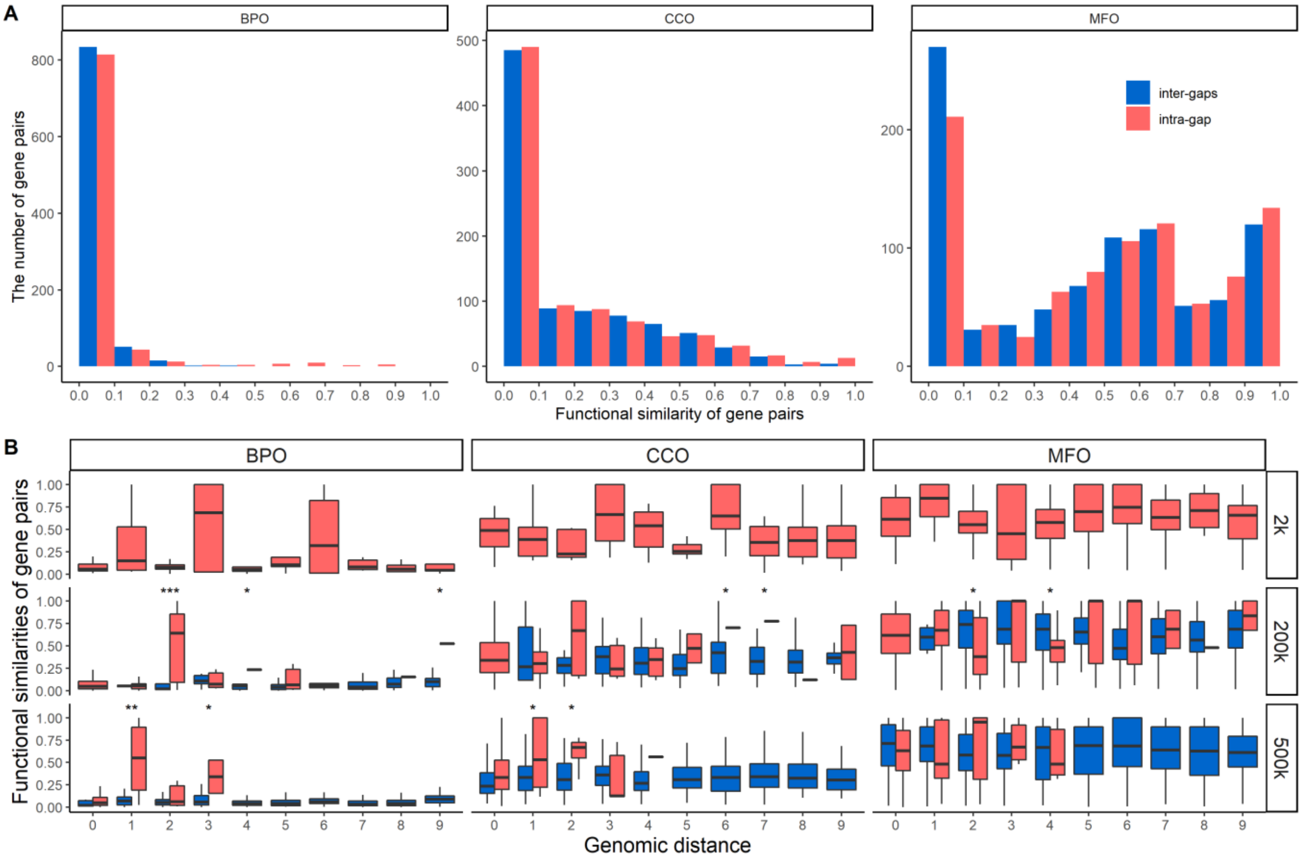

Supplementary Figure S8 Functional similarities of human gene pairs for intra- and inter-gaps removing possible duplicate genes. Supplementary Figure S8A depicts the histograms of functional similarities of gene pairs in BPO, CCO, and MFO. Supplementary Figure S8B shows the functional similarities of gene pairs from a range of genomic distances: bin sizes of 2 kbp, 200 kbp, and 500 kbp. For example, when bin size equals 2 kbp, bin 0 indicates that the genomic distance of the gene pairs is less than 2 kbp. *** indicates that the p-value of the Wilcoxon test is less than 0.0001, ** indicates that the p-value is between 0.0001 and 0.001, and * indicates that the p-value is between 0.001 and 0.05.

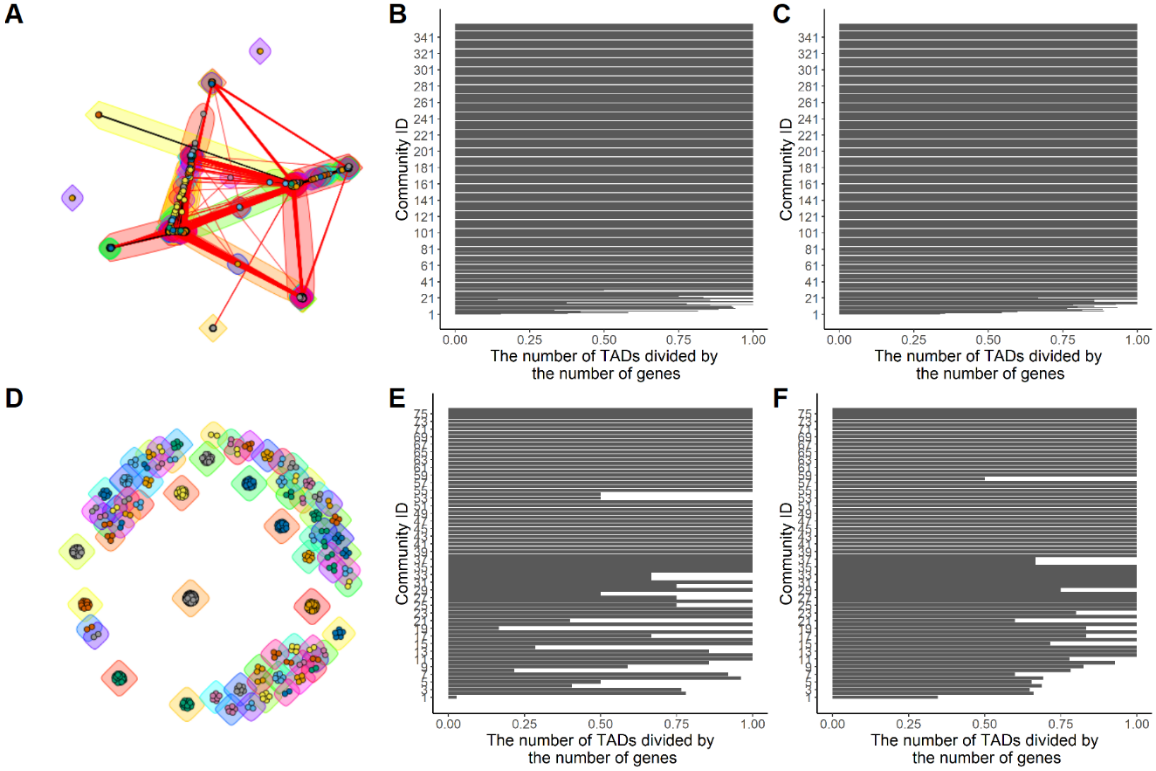

Supplementary Figure S9 The network communities for CCO FSNs of mouse chromosome 2. Figures A and D show the network communities with functional thresholds 0.7 and 1, respectively. Figures B and E show the distributions of the same-TAD-belonging ratios. Figures C and F show the distributions of the same-TAD-belonging ratios based on random communities.

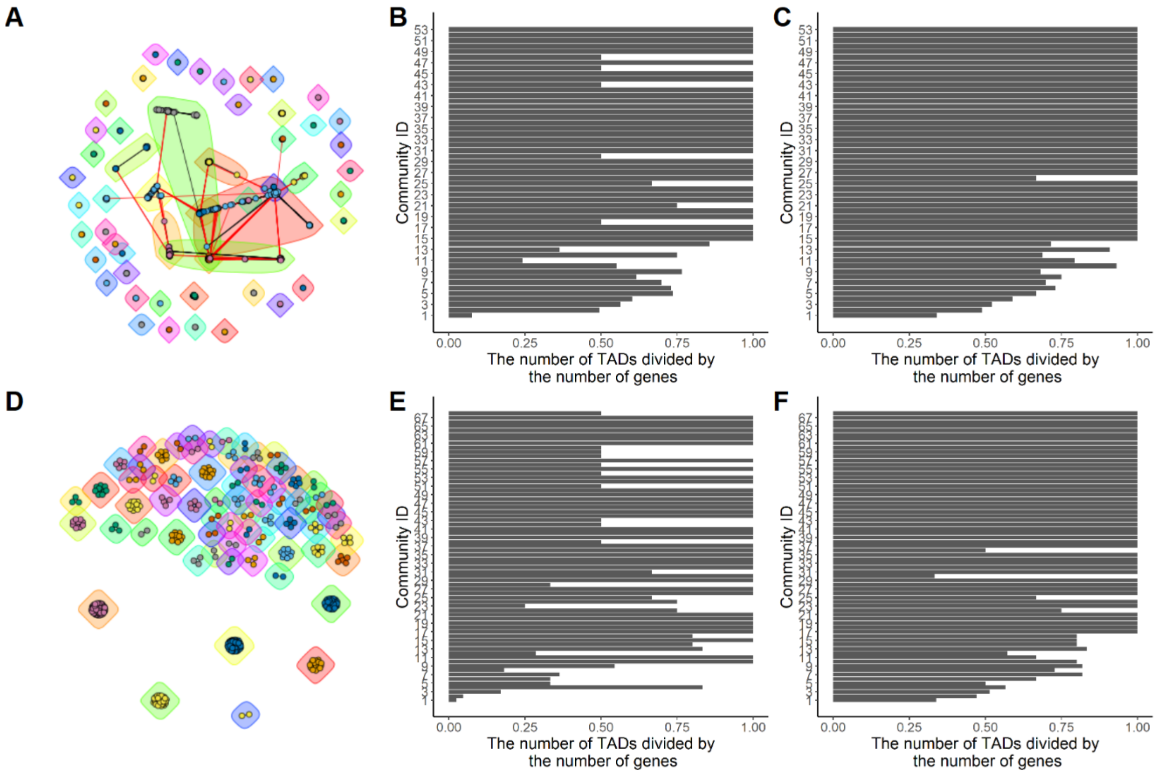

Supplementary Figure S10 The network communities for MFO FSNs of mouse chromosome 2. Figures A and D show the network communities with functional thresholds 0.7 and 1, respectively. Figures B and E show the distributions of the same-TAD-belonging ratios. Figures C and F show the distributions of the same-TAD-belonging ratios based on random communities.

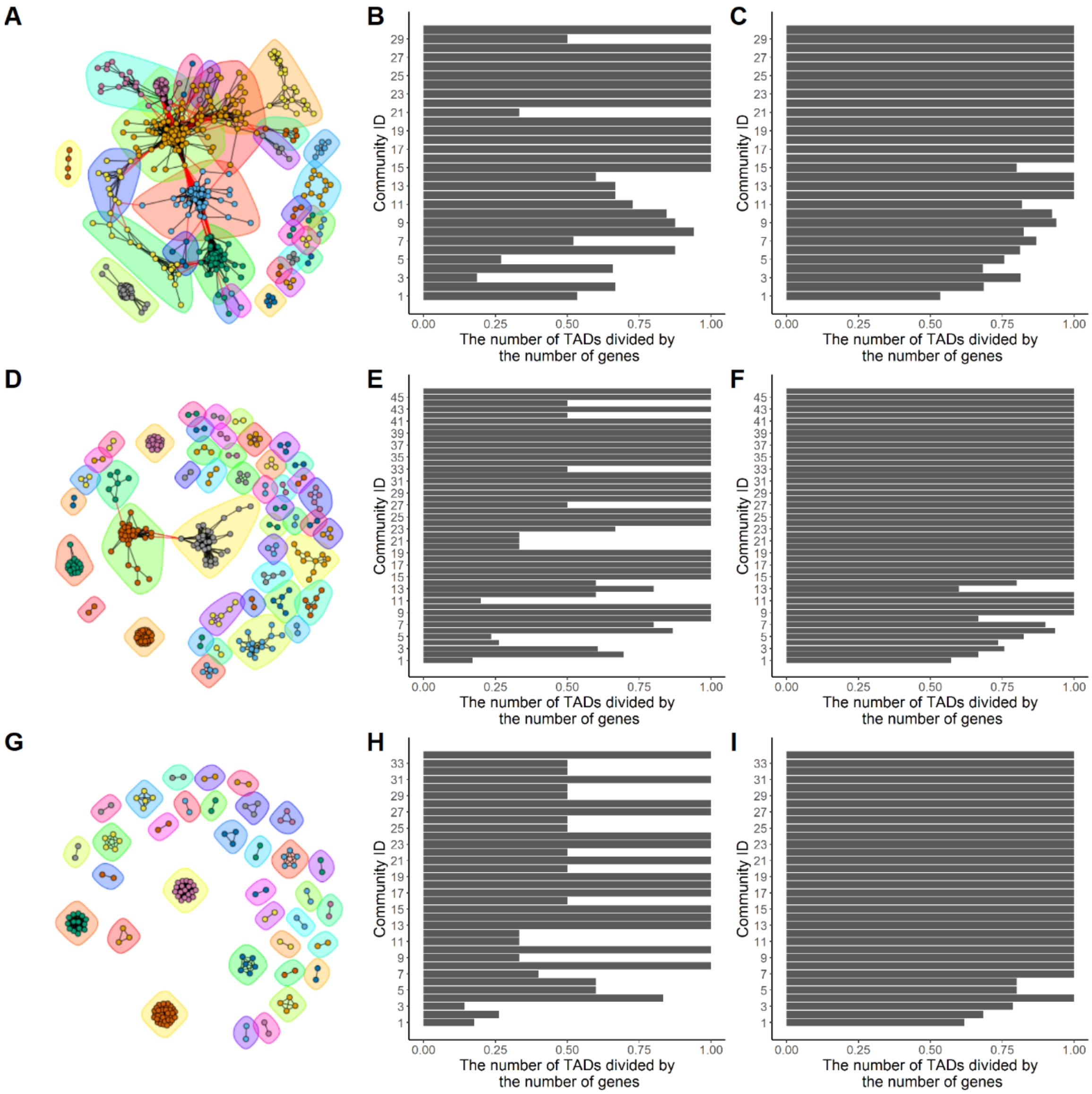

Supplementary Figure S11 The network communities for BPO FSNs of the mouse X-chromosome. Figures A, D, and G show the network communities with functional thresholds 0.5, 0.7, and 1, respectively. Figures B, E, and H show the distributions of the same-TAD-belonging ratios. Figures C, F, and I show the distributions of the same-TAD-belonging ratios based on random communities.

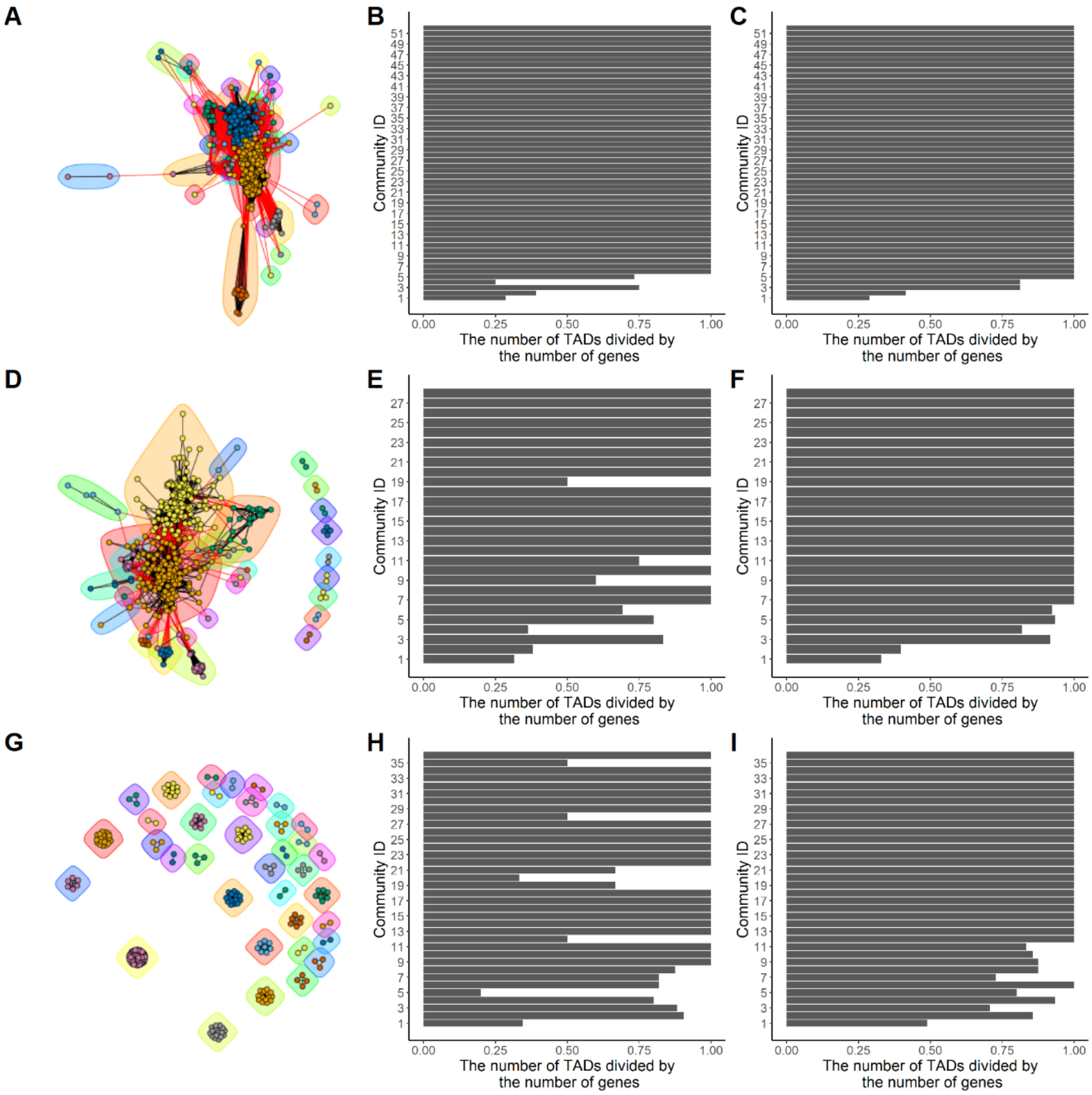

Supplementary Figure S12 The network communities for CCO FSNs of the mouse X-chromosome. Figures A, D, and G show the network communities with functional thresholds 0.5, 0.7, and 1, respectively. Figures B, E, and H show the distributions of the same-TAD-belonging ratios. Figures C, F, and I show the distributions of the same-TAD-belonging ratios based on random communities.

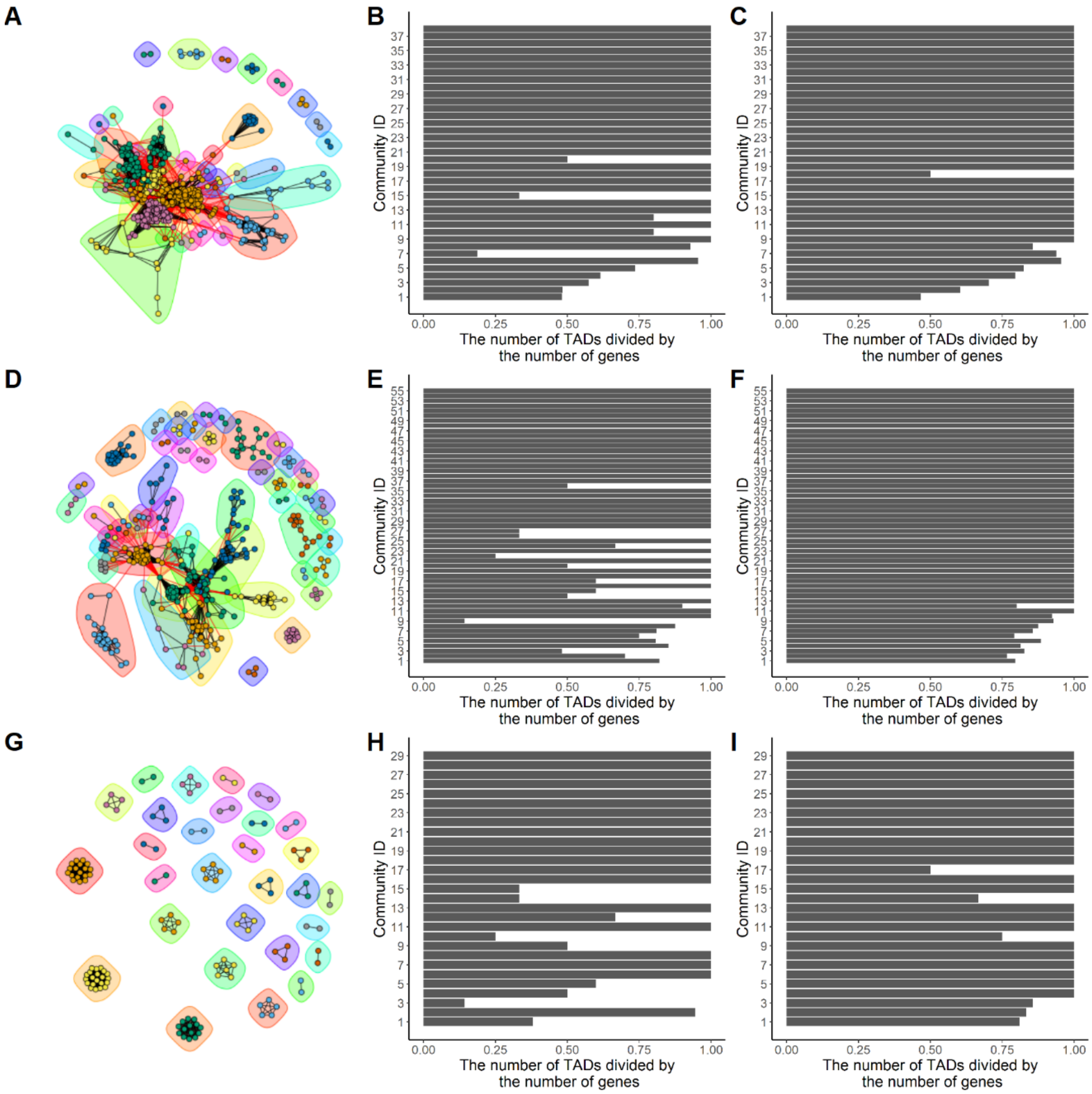

Supplementary Figure S13 The network communities for MFO FSNs of the mouse X-chromosome. Figures A, D, and G show the network communities with functional thresholds 0.5, 0.7, and 1, respectively. Figures B, E, and H show the distributions of the same-TAD-belonging ratios. Figures C, F, and I show the distributions of the same-TAD-belonging ratios based on random communities.

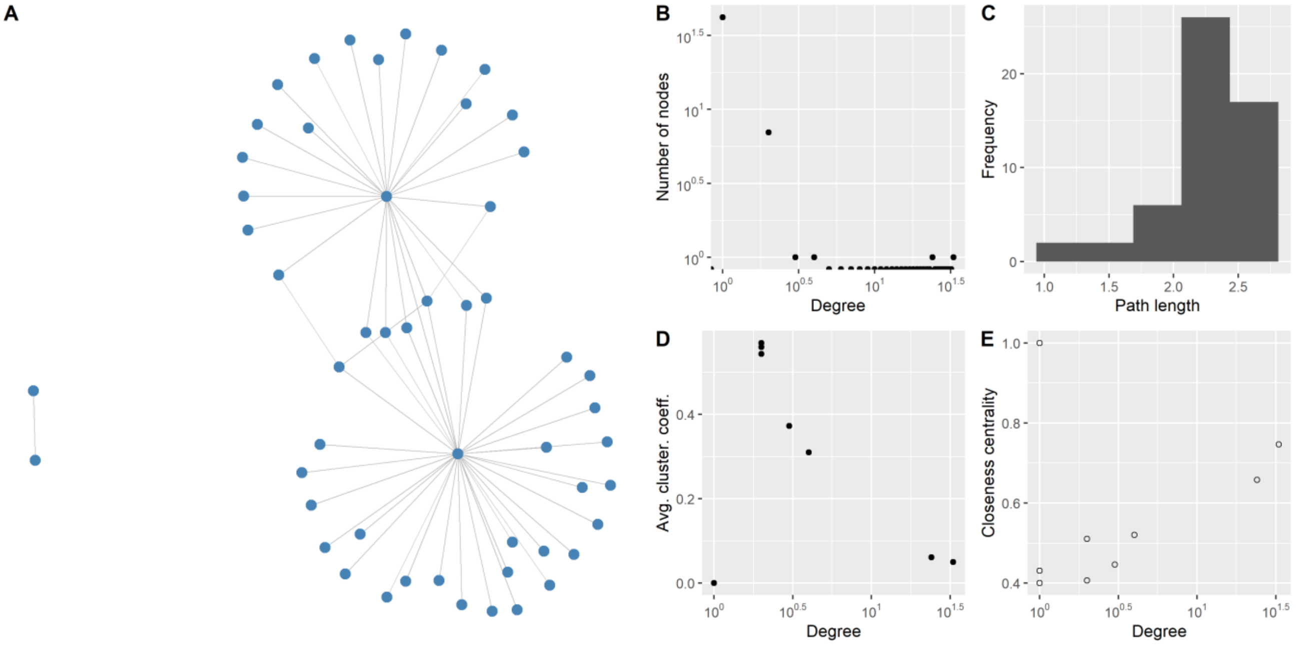

Supplementary Figure S14 The HiC-GGSI network and topological properties of the mouse X-chromosome with the Hi-C threshold: 800 and the distance threshold: 2 Mbp. Figure A represents the HiC-GGSI network. Figures B, C, D, and E represent the distributions of node degree, the average shortest path length, average cluster coefficient, and closeness centrality, respectively.

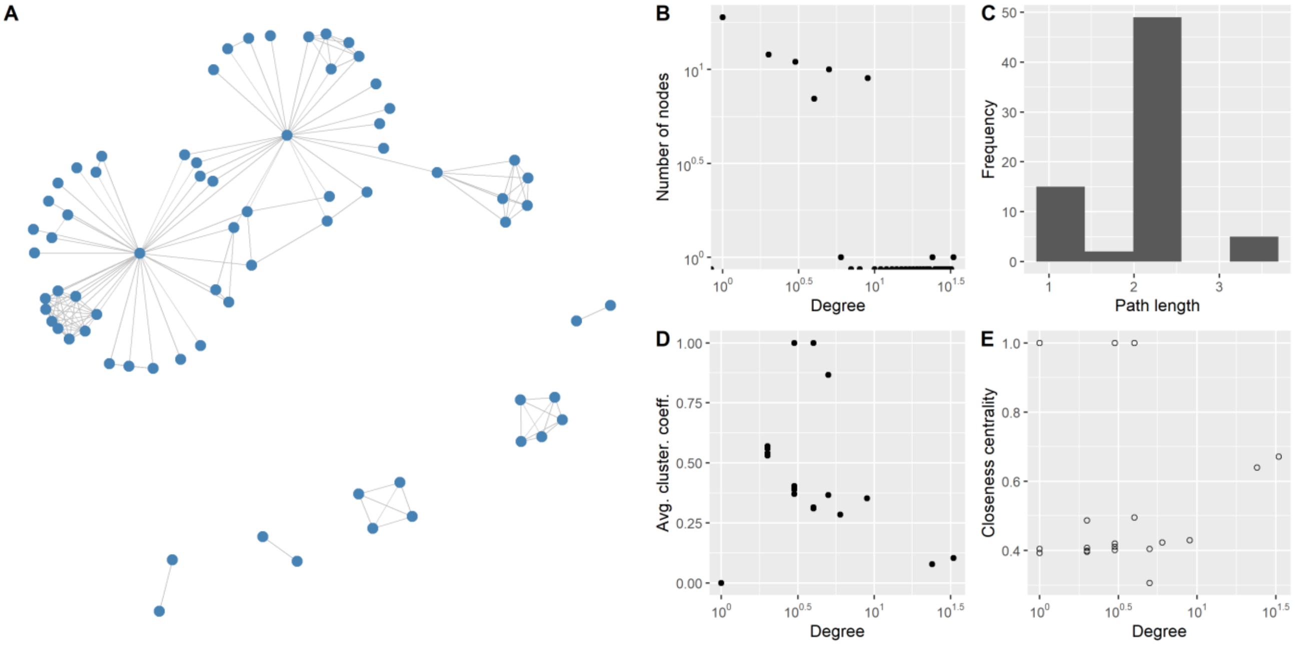

Supplementary Figure S15 The HiC-TAD-GGSI network and topological properties of the mouse X-chromosome with the Hi-C threshold: 800 and the distance threshold: 2 Mbp. Figure A represents the HiC-TAD-GGSI network. Figures B, C, D, and E represent the distributions of node degree, the average shortest path length, average cluster coefficient, and closeness centrality, respectively.

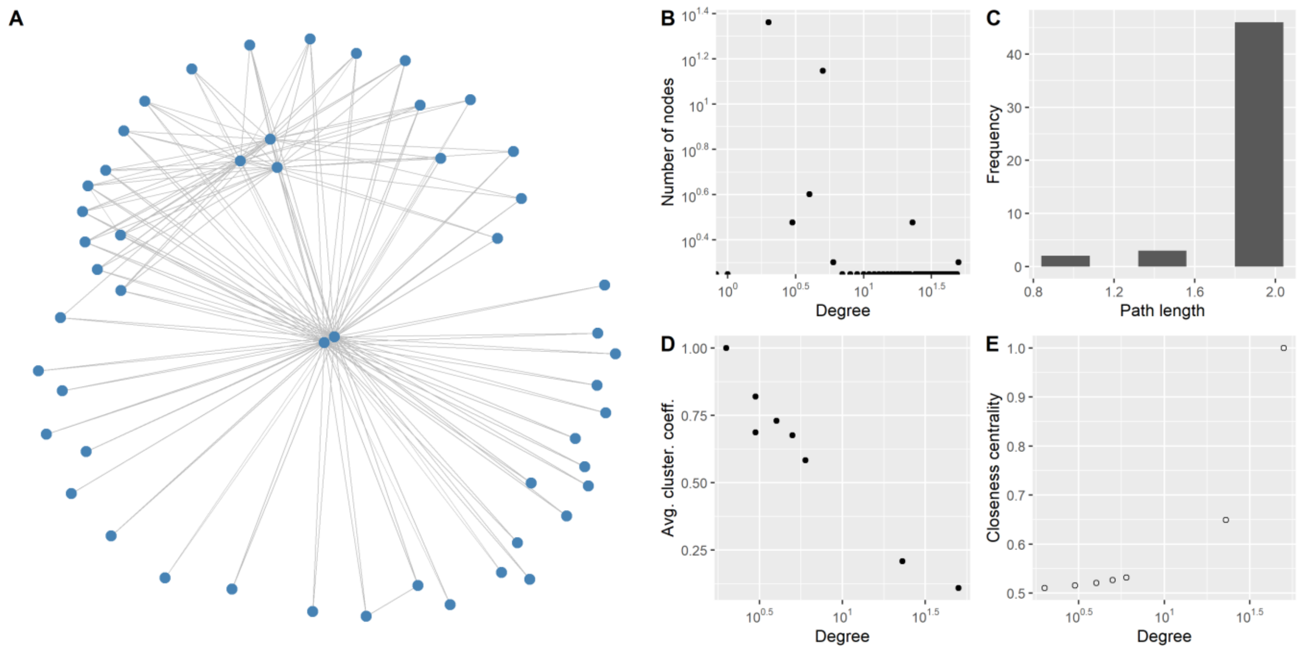

Supplementary Figure S16 The reconstructed HiC-GGSI network and topological properties of the mouse X-chromosome with the Hi-C threshold: 800, the distance threshold: 2 Mbp, and the autoencoder confidence score threshold: 0.6. Figure A represents the imputed HiC-GGSI network. Figures B, C, D, and E represent the distributions of node degree, the average shortest path length, average cluster coefficient, and closeness centrality, respectively.

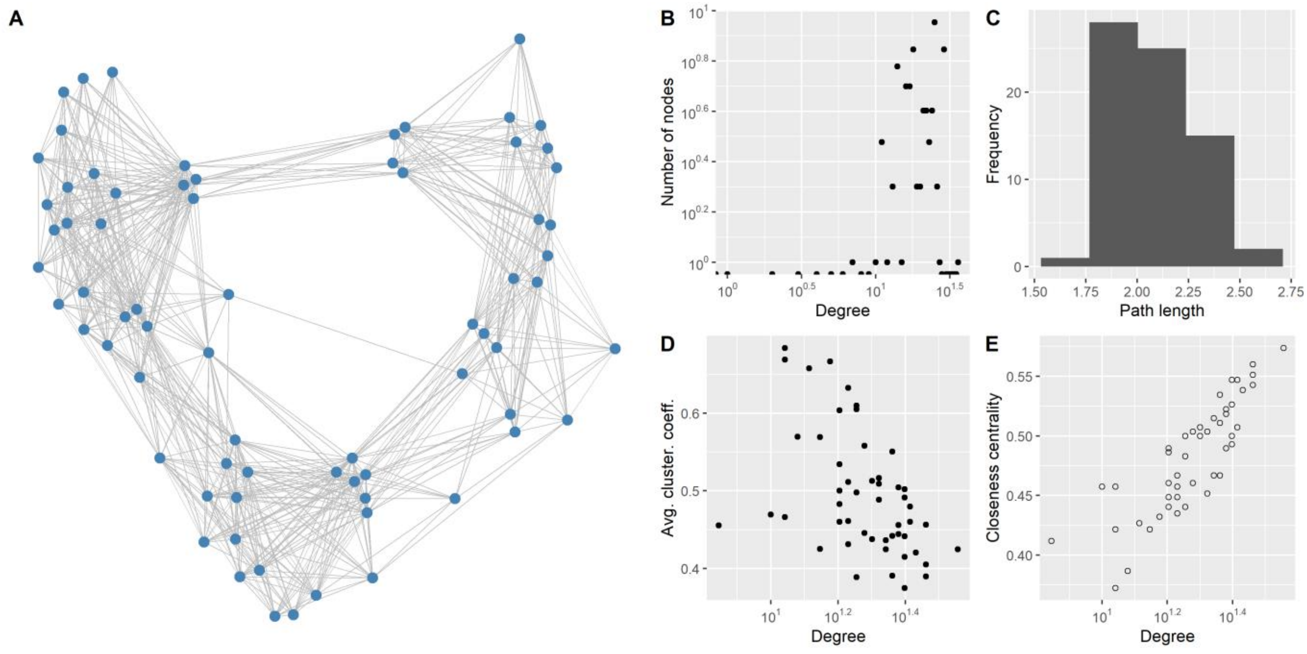

Supplementary Figure S17 The reconstructed HiC-TAD-GGSI network and topological properties of the mouse X-chromosome with the Hi-C threshold: 800, the distance threshold: 2 Mbp, and the autoencoder confidence score threshold: 0.6. Figure A represents the imputed HiC-TAD-GGSI

network. Figures B, C, D, and E represent the distributions of node degree, the average shortest path length, average cluster coefficient, and closeness centrality, respectively.

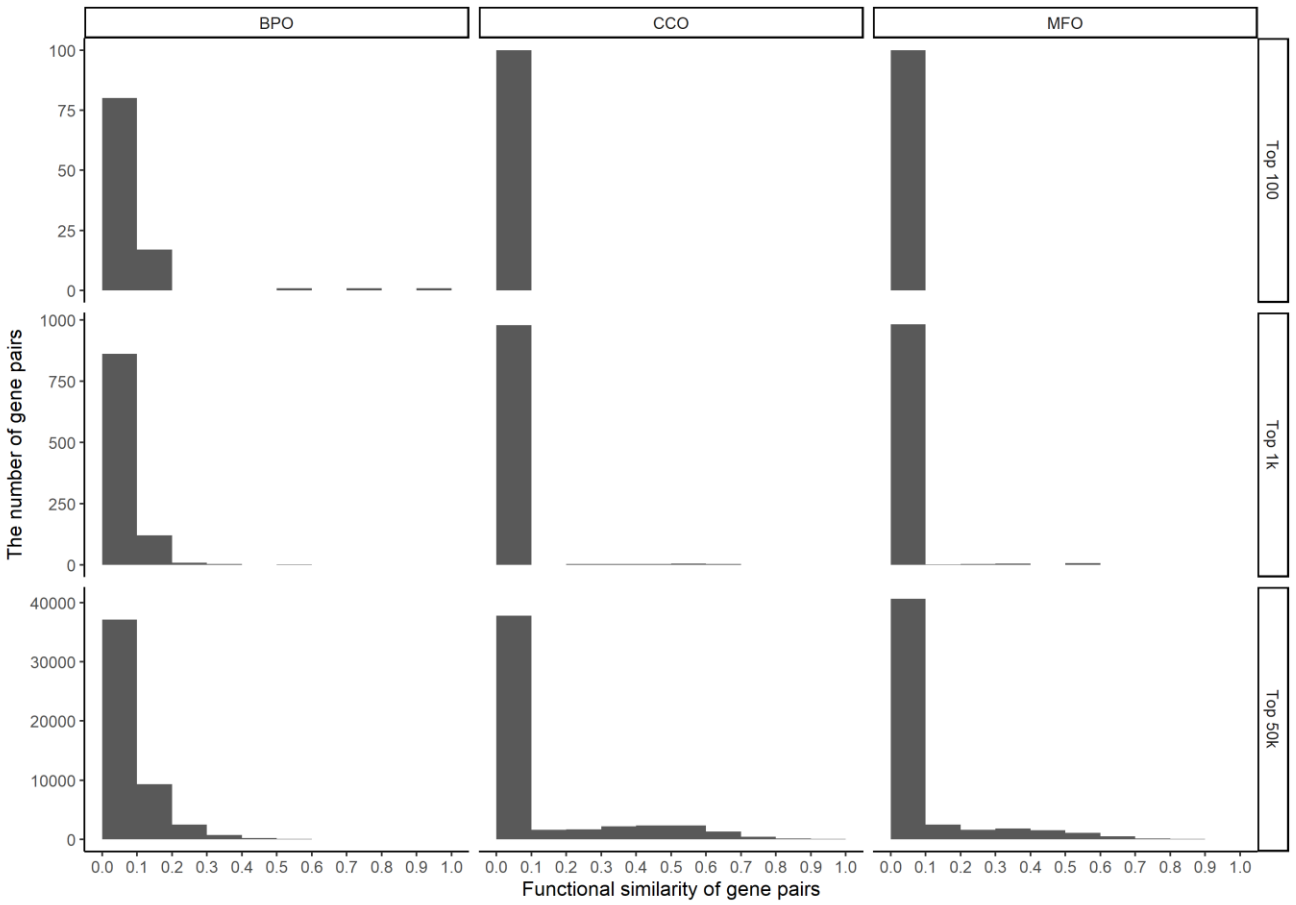

Supplementary Figure S18 Functional similarities of highly interactive mouse gene pairs from different chromosomes. The histograms of functional similarities of the gene pairs were calculated in BPO, CCO, and MFO. The gene pairs were sorted by the Hi-C inter-chromosome raw contacts. We plotted the histograms for the top 100, 1k, and 50k interactive gene pairs.

2 Supplementary Tables

Supplementary Table S1 The top 20 enriched gene functions associated with the high functional similarities (≥0.9) of intra-TADs gene pairs of humans and mice in BPO. The conserved gene functions across humans and mice are marked in bold.

Enriched gene functions of humans	Enriched gene functions of mice
**detection of chemical stimulus involved in sensory perception of bitter taste (GO:0001580)**	sensory perception of smell (GO:0007608)
sensory perception of bitter taste (GO:0050913)	xenobiotic glucuronidation (GO:0052697)
detection of chemical stimulus involved in sensory perception of taste (GO:0050912)	flavonoid glucuronidation (GO:0052696)
antibacterial humoral response (GO:0019731)	sensory perception of chemical stimulus (GO:0007606)
sensory perception of taste (GO:0050909)	cellular glucuronidation (GO:0052695)
antimicrobial humoral response (GO:0019730)	flavonoid metabolic process (GO:0009812)
cellular process (GO:0009987)	response to pheromone (GO:0019236)
biological regulation (GO:0065007)	uronic acid metabolic process (GO:0006063)
regulation of biological process (GO:0050789)	glucuronate metabolic process (GO:0019585)
regulation of cellular process (GO:0050794)	sensory perception (GO:0007600)
localization (GO:0051179)	response to corticosterone (GO:0051412)

regulation of metabolic process (GO:0019222)	G protein-coupled receptor signaling pathway (GO:0007186)
developmental process (GO:0032502)	nervous system process (GO:0050877)
regulation of macromolecule metabolic process (GO:0060255)	complement receptor mediated signaling pathway (GO:0002430)
regulation of cellular metabolic process (GO:0031323)	detection of chemical stimulus involved in sensory perception of smell (GO:0050911)
regulation of nitrogen compound metabolic process (GO:0051171)	detection of chemical stimulus involved in sensory perception (GO:0050907)
anatomical structure development (GO:0048856)	granzyme-mediated programmed cell death signaling pathway (GO:0140507)
regulation of primary metabolic process (GO:0080090)	cytolysis (GO:0019835)
regulation of cellular macromolecule biosynthetic process (GO:2000112)	system process (GO:0003008)
regulation of macromolecule biosynthetic process (GO:0010556)	**detection of chemical stimulus involved in sensory perception of bitter taste (GO:0001580)**

Supplementary Table S2 The top 20 enriched gene functions associated with the high functional similarities (≥0.9) of intra-TADs gene pairs of humans and mice in CCO. The conserved gene functions across humans and mice are marked in bold.

Enriched gene functions of humans	Enriched gene functions of mice
**chromatin (GO:0000785)**	CENP-A containing nucleosome (GO:0043505)
nucleoplasm (GO:0005654)	CENP-A containing chromatin (GO:0061638)
nuclear lumen (GO:0031981)	chromosome, centromeric core domain (GO:0034506)
chromosome (GO:0005694)	cytolytic granule (GO:0044194)
organelle lumen (GO:0043233)	MHC class Ib protein complex (GO:0032398)
intracellular organelle lumen (GO:0070013)	nucleosome (GO:0000786)
membrane-enclosed lumen (GO:0031974)	DNA packaging complex (GO:0044815)
nucleus (GO:0005634)	**integral component of membrane (GO:0016021)**
intracellular membrane-bounded organelle (GO:0043231)	**intrinsic component of membrane (GO:0031224)**
membrane-bounded organelle (GO:0043227)	**membrane (GO:0016020)**
intracellular organelle (GO:0043229)	**cellular anatomical entity (GO:0110165)**
organelle (GO:0043226)	plasma membrane (GO:0005886)
intracellular anatomical structure (GO:0005622)	extracellular space (GO:0005615)
**cellular anatomical entity (GO:0110165)**	cell periphery (GO:0071944)

**membrane (GO:0016020)**	extracellular region (GO:0005576)
cell periphery (GO:0071944)	external side of plasma membrane (GO:0009897)
**intrinsic component of membrane (GO:0031224)**	lytic vacuole (GO:0000323)
**integral component of membrane (GO:0016021)**	lysosome (GO:0005764)
organelle membrane (GO:0031090)	**chromatin (GO:0000785)**
plasma membrane bounded cell projection (GO:0120025)	side of membrane (GO:0098552)

Supplementary Table S3 The top 20 enriched gene functions associated with the high functional similarities (≥0.9) of intra-TADs gene pairs of humans and mice in MFO. The conserved gene functions across humans and mice are marked in bold.

Enriched gene functions of humans	Enriched gene functions of mice
**bitter taste receptor activity (GO:0033038)**	cholesterol dehydrogenase activity (GO:0102294)
protein binding (GO:0005515)	palmitoyl-CoA 9-desaturase activity (GO:0032896)
binding (GO:0005488)	olfactory receptor activity (GO:0004984)
molecular_function (GO:0003674)	odorant binding (GO:0005549)
heterocyclic compound binding (GO:1901363)	CCR1 chemokine receptor binding (GO:0031726)
cation binding (GO:0043169)	pheromone receptor activity (GO:0016503)
organic cyclic compound binding (GO:0097159)	pheromone binding (GO:0005550)
ion binding (GO:0043167)	trace-amine receptor activity (GO:0001594)
hydrolase activity (GO:0016787)	TAP2 binding (GO:0046979)
catalytic activity (GO:0003824)	TAP1 binding (GO:0046978)
purine ribonucleoside triphosphate binding (GO:0035639)	TAP binding (GO:0046977)
purine nucleotide binding (GO:0017076)	anandamide 11,12 epoxidase activity (GO:0062188)

purine ribonucleotide binding (GO:0032555)	anandamide 8,9 epoxidase activity (GO:0062187)
carbohydrate derivative binding (GO:0097367)	**bitter taste receptor activity (GO:0033038)**
ribonucleotide binding (GO:0032553)	caffeine oxidase activity (GO:0034875)
small molecule binding (GO:0036094)	oxidoreductase activity, acting on CH or CH2 groups, quinone or similar compound as acceptor (GO:0033695)
catalytic activity, acting on a protein (GO:0140096)	CD8 receptor binding (GO:0042610)
nucleotide binding (GO:0000166)	natural killer cell lectin-like receptor binding (GO:0046703)
nucleoside phosphate binding (GO:1901265)	transmembrane signaling receptor activity (GO:0004888)
cytoskeletal protein binding (GO:0008092)	anandamide 14,15 epoxidase activity (GO:0062189)

Supplementary Table S4 GO term enrichment analysis for gene pairs with high expression similarities. Gene pairs that have both the means of normalized expression counts >1000 and expression similarity scores >0.95 were used to generate this table. We did not find enriched GO terms for the genes from the intra-gap regions.

Genes observed from	Gene ontology	GO term name	Expected	Fold enrichment	Raw p-value
Inter-TADs	BPO	negative regulation of calcium ion export across plasma membrane (GO:1905913)	0.02	92.78	6.70E-04
		positive regulation of stress granule assembly (GO:0062029)	0.02	92.78	6.70E-04
		positive regulation of telomerase RNA localization to Cajal body (GO:1904874)	0.04	69.58	4.06E-05
	MFO	N-terminal myristoylation domain binding (GO:0031997)	0.03	92.78	2.34E-05
		adenylate cyclase activator activity (GO:0010856)	0.05	55.67	6.45E-05
		protein phosphatase activator activity (GO:0072542)	0.13	30.93	2.07E-05
	CCO	proteasome storage granule (GO:0034515)	0.02	92.78	6.70E-04
		box H/ACA telomerase RNP complex (GO:0090661)	0.04	46.39	1.65E-03
		proteasome core complex, alpha-subunit complex (GO:0019773)	0.09	46.39	5.84E-06
Inter-gaps	BPO	positive regulation of establishment of protein localization to telomere (GO:1904851)	0.02	>100	9.52E-07
		positive regulation of telomere maintenance via telomerase (GO:0032212)	0.05	60.63	2.13E-05
		toxin transport (GO:1901998)	0.07	43.11	5.55E-05
	MFO	protein folding chaperone (GO:0044183)	0.06	53.89	2.96E-05
		RNA binding (GO:0003723)	1.75	7.41	6.46E-09

	CCO	chaperonin-containing T-complex (GO:0005832)	0.02	>100	3.51E-04
		zona pellucida receptor complex (GO:0002199)	0.02	>100	1.86E-06
		myelin sheath (GO:0043209)	0.33	15.04	2.09E-05
Intra-TADs	BPO	de novo' IMP biosynthetic process (GO:0006189)	0.02	>100	2.59E-07
		de novo' AMP biosynthetic process (GO:0044208)	0.04	>100	2.55E-04
		ribosomal small subunit export from nucleus (GO:0000056)	0.04	67.86	2.42E-05
	MFO	ceramide binding (GO:0097001)	0.09	32.15	1.63E-04
		aminoacyl-tRNA ligase activity (GO:0004812)	0.2	19.86	6.89E-05
		structural constituent of cytoskeleton (GO:0005200)	0.31	15.91	2.23E-05
	CCO	nucleoplasmic periphery of the nuclear pore complex (GO:1990826)	0.01	>100	1.41E-04
		PTW/PP1 phosphatase complex (GO:0072357)	0.03	58.17	8.33E-04
		annulate lamellae (GO:0005642)	0.03	58.17	8.33E-04

Supplementary Tables S5 Mutual pathways for gene pairs with high expression similarities. Gene pairs that have both the means of normalized expression counts >1000 and expression similarity scores >0.95 were used to generate this table. We did not find any mutual pathway for the gene pairs from intra- and inter-gap regions.

Genes observed from	Gene1	Gene2	Mutual pathways
Inter-TADs	MGI:107686	MGI:1298388	Metabolic pathways (mmu01100)
	MGI:98890	MGI:1913284	Pathways of neurodegeneration (mmu05022)
	MGI:98890	MGI:1913284	Parkinson disease (mmu05012)
	MGI:98890	MGI:1920150	Pathways of neurodegeneration (mmu05022)
	MGI:98890	MGI:1920150	Parkinson disease (mmu05012)
	MGI:1913663	MGI:107686	Alzheimer disease (mmu05010)
	MGI:1913663	MGI:107686	Pathways of neurodegeneration (mmu05022)
	MGI:1913663	MGI:107686	Prion disease (mmu05020)
	MGI:1913663	MGI:107686	Huntington disease (mmu05016)
	MGI:1913663	MGI:107686	Amyotrophic lateral sclerosis (mmu05014)
	MGI:1913663	MGI:107686	Parkinson disease (mmu05012)
Intra-TAD	MGI:1351455	MGI:1351329	Ribosome (mmu03010)
	MGI:1351455	MGI:1351329	Coronavirus disease (mmu05171)
	MGI:1914693	MGI:88473	Pathways of neurodegeneration (mmu05022)
	MGI:1914693	MGI:88473	Amyotrophic lateral sclerosis (mmu05014)
	MGI:97549	MGI:104880	Amyotrophic lateral sclerosis (mmu05014)
	MGI:98869	MGI:1095409	Phagosome (mmu04145)
	MGI:98869	MGI:1095409	Alzheimer disease (mmu05010)
	MGI:98869	MGI:1095409	Prion disease (mmu05020)
	MGI:98869	MGI:1095409	Pathways of neurodegeneration (mmu05022)
	MGI:98869	MGI:1095409	Salmonella infection (mmu05132)
	MGI:98869	MGI:1095409	Huntington disease (mmu05016)
	MGI:98869	MGI:1095409	Gap junction (mmu04540)
	MGI:98869	MGI:1095409	Amyotrophic lateral sclerosis (mmu05014)
	MGI:98869	MGI:1095409	Parkinson disease (mmu05012)
	MGI:98869	MGI:1095409	Tight junction (mmu04530)
	MGI:98869	MGI:1095409	Apoptosis (mmu04210)
	MGI:107804	MGI:1095409	Phagosome (mmu04145)

	MGI:107804	MGI:1095409	Alzheimer disease (mmu05010)
	MGI:107804	MGI:1095409	Prion disease (mmu05020)
	MGI:107804	MGI:1095409	Pathways of neurodegeneration (mmu05022)
	MGI:107804	MGI:1095409	Salmonella infection (mmu05132)
	MGI:107804	MGI:1095409	Huntington disease (mmu05016)
	MGI:107804	MGI:1095409	Gap junction (mmu04540)
	MGI:107804	MGI:1095409	Amyotrophic lateral sclerosis (mmu05014)
	MGI:107804	MGI:1095409	Parkinson disease (mmu05012)
	MGI:107804	MGI:1095409	Tight junction (mmu04530)
	MGI:107804	MGI:1095409	Apoptosis (mmu04210)
	MGI:107494	MGI:1914518	Focal adhesion (mmu04510)
	MGI:107494	MGI:1914518	Axon guidance (mmu04360)
	MGI:107494	MGI:1914518	Leukocyte transendothelial migration (mmu04670)
	MGI:107494	MGI:1914518	Salmonella infection (mmu05132)
	MGI:107494	MGI:1914518	Regulation of actin cytoskeleton (mmu04810)
	MGI:107494	MGI:1914518	Tight junction (mmu04530)
	MGI:107494	MGI:1914518	Platelet activation (mmu04611)

Supplementary Table S6 The performance of the graph autoencoder when it was used to reconstruct the HiC-GGSI and HiC-TAD-GGSI networks at different Hi-C contact and genomic distance thresholds on the chromosomes 2 of mice and humans and the chromosome 2A of chimpanzees. We calculated the 95% confidence interval with ten repeated experiments.

Species	Experimental settings			Number of genes in the network	The area under the curve (AUC)	Average precision (AP)
	Number of Hi-C contacts between gene pairs	Genomic distance between gene pairs	Network type			
Mouse	≥800	≥1 Mbp	HiC-GGSI	94	0.76±0.074	0.79±0.077
			HiC-TAD-GGSI	618	1.0±0.0	1.0±0.0
		≥2 Mbp	HiC-GGSI	57	0.86±0.089	0.91±0.055
			HiC-TAD-GGSI	113	0.96±0.019	0.97±0.013
	≥1200	≥1 Mbp	HiC-GGSI	61	0.7±0.165	0.75±0.114
			HiC-TAD-GGSI	591	1.0±0.001	1.0±0.001
		≥2 Mbp	HiC-GGSI	32	0.86±0.187	0.9±0.132
			HiC-TAD-GGSI	88	0.98±0.012	0.99±0.009
Human	≥5	≥1 Mbp	HiC-GGSI	191	0.83±0.022	0.87±0.021
			HiC-TAD-GGSI	200	0.83±0.03	0.87±0.024
		≥2 Mbp	HiC-GGSI	165	0.82±0.013	0.86±0.011
			HiC-TAD-GGSI	167	0.82±0.023	0.85±0.029
	≥10	≥1 Mbp	HiC-GGSI	116	0.76±0.049	0.82±0.029
			HiC-TAD-GGSI	129	0.83±0.047	0.85±0.042
		≥2 Mbp	HiC-GGSI	85	0.69±0.062	0.77±0.039
			HiC-TAD-GGSI	89	0.77±0.052	0.81±0.062
Chimpanzee	≥30	⩾1.5 Mbp	HiC-GGSI	150	0.83±0.035	0.86±0.026
			HiC-TAD-GGSI	161	0.86±0.028	0.9±0.019

		⩾2 Mbp	HiC-GGSI	132	0.85±0.04	0.88±0.029
			HiC-TAD-GGSI	134	0.84±0.031	0.88±0.025
	≥80	⩾1.5 Mbp	HiC-GGSI	77	0.68±0.087	0.74±0.08
			HiC-TAD-GGSI	89	0.78±0.062	0.83±0.074
		⩾2 Mbp	HiC-GGSI	66	0.69±0.077	0.75±0.047
			HiC-TAD-GGSI	69	0.73±0.074	0.79±0.072

Supplementary Table S7 Performance of function prediction based on reconstructed networks on the chromosomes 2 of mice and humans and the chromosome 2A of chimpanzees. We applied a threshold value of 0.6 to the autoencoder confidence scores. We calculated the 95% confidence interval with ten repeated experiments.

Species	Experimental settings			GO terms considered for evaluation	Average of the best functional similarity between true GO terms and the GO terms inferred from:		
	Number of Hi-C contacts between gene pairs	Genomic distance between gene pairs	Network type		original network	reconstructed network	union of original and reconstructed networks
Mouse	≥800	≥1 Mbp	HiC-GGSI	Top 1	0.41±0.0	0.23±0.097	0.42±0.003
				Top 4	0.63±0.0	0.5±0.184	0.64±0.004
			HiC-TAD-GGSI	Top 1	0.69±0.0	0.7±0.01	0.7±0.009
				Top 4	0.87±0.0	0.91±0.003	0.91±0.003
		≥2 Mbp	HiC-GGSI	Top 1	0.42±0.0	0.3±0.152	0.43±0.011
				Top 4	0.5±0.0	0.46±0.251	0.52±0.017
			HiC-TAD-GGSI	Top 1	0.53±0.0	0.6±0.046	0.58±0.036
				Top 4	0.7±0.0	0.85±0.07	0.81±0.067
	≥1200	≥1 Mbp	HiC-GGSI	Top 1	0.4±0.0	0.45±0.129	0.45±0.078
				Top 4	0.61±0.0	0.74±0.065	0.66±0.074
			HiC-TAD-GGSI	Top 1	0.7±0.0	0.69±0.008	0.69±0.007
				Top 4	0.88±0.0	0.9±0.004	0.9±0.007
		≥2 Mbp	HiC-GGSI	Top 1	0.35±0.0	0.43±0.112	0.37±0.046
				Top 4	0.48±0.0	0.73±0.115	0.52±0.084
			HiC-TAD-GGSI	Top 1	0.53±0.0	0.58±0.045	0.56±0.031
				Top 4	0.72±0.0	0.82±0.052	0.78±0.044
Human	≥5	≥1 Mbp	HiC-GGSI	Top 1	0.61±0.0	0.79±0.068	0.81±0.039
				Top 4	0.81±0.0	0.89±0.03	0.9±0.009

			HiC-TAD-GGSI	Top 1	0.66±0.0	0.83±0.0	0.83±0.0
				Top 4	0.82±0.0	0.91±0.0	0.91±0.0
		≥2 Mbp	HiC-GGSI	Top 1	0.64±0.0	0.78±0.052	0.77±0.072
				Top 4	0.84±0.0	0.89±0.016	0.89±0.014
			HiC-TAD-GGSI	Top 1	0.67±0.0	0.82±0.0	0.82±0.0
				Top 4	0.85±0.0	0.9±0.002	0.9±0.002
	≥10	≥1 Mbp	HiC-GGSI	Top 1	0.54±0.0	0.65±0.105	0.67±0.254
				Top 4	0.76±0.0	0.81±0.04	0.86±0.015
			HiC-TAD-GGSI	Top 1	0.59±0.0	0.82±0.0	0.82±0.0
				Top 4	0.79±0.0	0.89±0.001	0.89±0.001
		≥2 Mbp	HiC-GGSI	Top 1	0.53±0.0	0.73±0.055	0.74±0.062
				Top 4	0.78±0.0	0.83±0.016	0.84±0.006
			HiC-TAD-GGSI	Top 1	0.57±0.0	0.8±0.0	0.8±0.0
				Top 4	0.78±0.0	0.87±0.002	0.87±0.002
Chimpanzee	≥30	≥1.5 Mbp	HiC-GGSI	Top 1	0.46±0.0	0.55±0.041	0.62±0.066
				Top 4	0.67±0.0	0.75±0.049	0.78±0.052
			HiC-TAD-GGSI	Top 1	0.53±0.0	0.56±0.015	0.59±0.035
				Top 4	0.71±0.0	0.78±0.027	0.79±0.008
		≥2 Mbp	HiC-GGSI	Top 1	0.46±0.0	0.53±0.043	0.59±0.02
				Top 4	0.69±0.0	0.75±0.039	0.78±0.035
			HiC-TAD-GGSI	Top 1	0.52±0.0	0.59±0.022	0.6±0.015
				Top 4	0.71±0.0	0.79±0.021	0.8±0.015
	≥80	≥1.5 Mbp	HiC-GGSI	Top 1	0.42±0.0	0.48±0.061	0.56±0.048

				Top 4	0.62±0.0	0.67±0.047	0.7±0.083
			HiC-TAD-GGSI	Top 1	0.44±0.0	0.47±0.032	0.47±0.088
				Top 4	0.63±0.0	0.66±0.029	0.71±0.041
		≥2 Mbp	HiC-GGSI	Top 1	0.4±0.0	0.42±0.038	0.57±0.103
				Top 4	0.65±0.0	0.66±0.029	0.78±0.232
			HiC-TAD-GGSI	Top 1	0.4±0.0	0.43±0.047	0.48±0.12
				Top 4	0.66±0.0	0.67±0.024	0.65±0.119

Supplementary Table S8 Functionally similar gene pairs from mouse long-range highly interactive regions with functional similarities >=0.5.

Gene pair distance threshold >=	Ontology	Chromosome	Functionally similar gene pairs from long-range highly interactive regions (similarity >=0.5)
3.2 Mbp	CCO	3	MGI:101784-MGI:3643869
	CCO	4	MGI:1916027-MGI:1913704
	CCO	5	MGI:1891679-MGI:101909
			MGI:97401-MGI:102851
			MGI:108059-MGI:101909
			MGI:1095413-MGI:2385237
			MGI:97401-MGI:88361
	CCO	6	MGI:97946-MGI:95700
	CCO	7	MGI:98783-MGI:2447816
			MGI:1924258-MGI:2447816
			MGI:104605-MGI:2447816
			MGI:2149590-MGI:2447816
	MFO	9	MGI:1933847-MGI:3648996
	BPO	10	MGI:3588256-MGI:3649078
	CCO	10	MGI:1861032-MGI:103579
			MGI:1861032-MGI:1351911
			MGI:1861032-MGI:99549
			MGI:3588256-MGI:3649078
	MFO	10	MGI:1861032-MGI:106618

			MGI:1861032-MGI:1918959<br>MGI:3588256-MGI:3649078
	CCO	13	MGI:97618-MGI:1913472
	CCO	15	MGI:3580656-MGI:109440
	CCO	17	MGI:98643-MGI:98535<br>MGI:3646674-MGI:95936<br>MGI:3646674-MGI:95935
	MFO	17	MGI:98643-MGI:3525201<br>MGI:98643-MGI:104798<br>MGI:98643-MGI:1345189<br>MGI:98643-MGI:104797<br>MGI:98643-MGI:3042141<br>MGI:98643-MGI:97603<br>MGI:98643-MGI:1354739<br>MGI:98643-MGI:1100500<br>MGI:98643-MGI:1101058<br>MGI:98643-MGI:98535<br>MGI:98643-MGI:1341188<br>MGI:98643-MGI:98352<br>MGI:98643-MGI:2443284
6.4 Mbp	CCO	4	MGI:1916027-MGI:1913704
	CCO	5	MGI:108059-MGI:101909
	CCO	6	MGI:97946-MGI:95700

	CCO	7	MGI:98783-MGI:2447816<br>MGI:1924258-MGI:2447816<br>MGI:104605-MGI:2447816<br>MGI:2149590-MGI:2447816
	MFO	9	MGI:1933847-MGI:3648996
	BPO	10	MGI:3588256-MGI:3649078
	CCO	10	MGI:1861032-MGI:103579<br>MGI:1861032-MGI:1351911<br>MGI:1861032-MGI:99549<br>MGI:3588256-MGI:3649078
	MFO	10	MGI:1861032-MGI:106618<br>MGI:1861032-MGI:1918959<br>MGI:3588256-MGI:3649078
	CCO	13	MGI:97618-MGI:1913472
	CCO	15	MGI:3580656-MGI:109440
	CCO	17	MGI:98643-MGI:98535<br>MGI:3646674-MGI:95936<br>MGI:3646674-MGI:95935
	MFO	17	MGI:98643-MGI:3525201<br>MGI:98643-MGI:104798<br>MGI:98643-MGI:1345189<br>MGI:98643-MGI:104797<br>MGI:98643-MGI:3042141

			MGI:98643-MGI:1354739<br>MGI:98643-MGI:1100500<br>MGI:98643-MGI:1101058<br>MGI:98643-MGI:98535<br>MGI:98643-MGI:1341188<br>MGI:98643-MGI:98352<br>MGI:98643-MGI:2443284
9.6 Mbp	CCO	4	MGI:1916027-MGI:1913704
	CCO	5	MGI:108059-MGI:101909
	MFO	9	MGI:1933847-MGI:3648996
	BPO	10	MGI:3588256-MGI:3649078
	CCO	10	MGI:1861032-MGI:103579<br>MGI:1861032-MGI:1351911<br>MGI:1861032-MGI:99549<br>MGI:3588256-MGI:3649078
	MFO	10	MGI:1861032-MGI:106618<br>MGI:1861032-MGI:1918959<br>MGI:3588256-MGI:3649078
	CCO	13	MGI:97618-MGI:1913472
	CCO	15	MGI:3580656-MGI:109440
	CCO	17	MGI:3646674-MGI:95936<br>MGI:3646674-MGI:95935

	MFO	17	MGI:98643-MGI:3525201<br>MGI:98643-MGI:104798<br>MGI:98643-MGI:1345189<br>MGI:98643-MGI:104797<br>MGI:98643-MGI:3042141<br>MGI:98643-MGI:1354739<br>MGI:98643-MGI:1100500<br>MGI:98643-MGI:1101058<br>MGI:98643-MGI:1341188<br>MGI:98643-MGI:2443284
40 Mbp	MFO	9	MGI:1933847-MGI:3648996
	CCO	10	MGI:1861032-MGI:103579<br>MGI:1861032-MGI:99549
	MFO	10	MGI:1861032-MGI:106618<br>MGI:1861032-MGI:1918959
	MFO	17	MGI:98643-MGI:1101058<br>MGI:98643-MGI:1345189

Supplementary Table S9 GO terms enrichment analysis of genes from long-range highly interactive regions. In Figure 5, when the genomic distance threshold was 3.2 Mbp, long-range highly interactive regions with significantly high average function similarities were observed in chromosomes 10 and 17. Enrichment analysis was applied to the genes from these regions. The genes from chromosome 10 do not have an enriched GO term.

Chromosome	Gene Ontology	GO term name	Expected	Fold enrichment	Raw p-value
17	MFO	acetyl-CoA C-acetyltransferase activity (GO:0003985)	0.01	>100	8.94E-05
		N-formyl peptide receptor activity (GO:0004982)	0.01	>100	1.15E-04
		RAGE receptor binding (GO:0050786)	0.02	>100	2.10E-04

Supplementary Table S10 Mutual pathways of gene pairs from long-range highly interactive regions. In Figure 5, when the gene ontology was MFO, and the genomic distance threshold was 3.2 Mbp, long-range highly interactive regions with significantly high average function similarities were observed in chromosomes 10 and 17. Mutual pathways were found for the genes from these regions. The genes from chromosome 10 do not have a mutual pathway.

Chromosome	Gene1	Gene2	Mutual pathways
17	MGI:98643	MGI:104798	Salmonella infection (mmu05132)
	MGI:98643	MGI:107812	Salmonella infection (mmu05132)

Supplementary Table S11 The mutual pathways of mouse inter-chromosome interactive gene pairs. Gene pairs were sorted by the number of Hi-C raw contacts. The top 7,000 ranked gene pairs were searched for the mutual pathways.

Gene 1	Chromosome of gene1	Gene 2	Chromosome of gene 2	Number of Hi-C raw contact	Rank of Hi-C raw contact	Mutual pathways
MGI:2661445	10	MGI:109611	13	292	912	Arrhythmogenic right ventricular cardiomyopathy (mmu05412)
MGI:1346525	11	MGI:109611	13	210	1666	Arrhythmogenic right ventricular cardiomyopathy (mmu05412)
MGI:109611	13	MGI:1338890	14	168	2446	Arrhythmogenic right ventricular cardiomyopathy (mmu05412)
MGI:88275	6	MGI:109611	13	158	2720	Arrhythmogenic right ventricular cardiomyopathy (mmu05412)
MGI:94909	X	MGI:109611	13	140	3274	Arrhythmogenic right ventricular cardiomyopathy (mmu05412)
MGI:103013	6	MGI:109611	13	114	4584	Arrhythmogenic right ventricular cardiomyopathy (mmu05412)
MGI:99912	10	MGI:109611	13	102	5441	Arrhythmogenic right ventricular cardiomyopathy (mmu05412)
MGI:1914047	6	MGI:1096389	12	100	5588	Cell adhesion molecules (mmu04514)
MGI:88295	5	MGI:109611	13	98	5739	Arrhythmogenic right ventricular cardiomyopathy (mmu05412)

MGI:95523	7	MGI:2661445	10	94	6110	Pathways in cancer (mmu05200),<br>Gastric cancer (mmu05226)
MGI:109611	13	MGI:88293	14	94	6116	Arrhythmogenic right ventricular cardiomyopathy (mmu05412)
MGI:95523	7	MGI:94869	18	92	6285	Pathways in cancer (mmu05200)
MGI:1354953	5	MGI:95523	7	86	6791	PI3K-Akt signaling pathway (mmu04151), Rap1 signaling pathway (mmu04015)
MGI:103013	6	MGI:95523	7	86	6879	MAPK signaling pathway (mmu04010), Calcium signaling pathway (mmu04020)
